# CCDC85A is regulated by miR-224-3p and augments cancer cell resistance to endoplasmic reticulum stress

**DOI:** 10.3389/fonc.2023.1196546

**Published:** 2023-07-18

**Authors:** So Takahashi, Kurara Takagane, Go Itoh, Sei Kuriyama, Michinobu Umakoshi, Akiteru Goto, Kazuyoshi Yanagihara, Masakazu Yashiro, Katsunori Iijima, Masamitsu Tanaka

**Affiliations:** ^1^ Department of Molecular Medicine and Biochemistry, Akita University Graduate School of Medicine, Akita, Japan; ^2^ Department of Gastroenterology, Akita University Graduate School of Medicine, Akita, Japan; ^3^ Department of Cellular and Organ Pathology, Akita University Graduate School of Medicine, Akita, Japan; ^4^ Division of Rare Cancer Research, National Cancer Center Research Institute, Tokyo, Japan; ^5^ Department of Surgical Oncology, Osaka City University Graduate School of Medicine, Osaka, Japan

**Keywords:** CCDC85A, miR224-3p, exosomes, ER stress, cisplatin resistance

## Abstract

MicroRNAs (miRNAs) play pivotal roles in the tumor microenvironment. Here, we analyzed miRNAs in tumor stromal fibroblasts. Expression of miR-224-3p in cancer-associated fibroblasts (CAF) from scirrhous gastric cancer patients was lower than in normal fibroblasts (NF). Introduction of a miR-224-3p mimic attenuated migration and invasion of CAF. Coiled-coil domain containing 85A (CCDC85A), whose function in tumors is not understood, was the target gene of miR-224-3p. Immunohistological analysis revealed that CCDC85A is expressed to varying degrees by cancer cells and CAFs in gastric and pancreatic carcinomas. Downregulation of CCDC85A in cancer cells revealed that these cells are vulnerable to endoplasmic reticulum (ER) stress induced by thapsigargin or tunicamycin, which were ameliorated after addback of CCDC85A. Injection of NF-derived exosomes containing miR-224-3p into the xenograft tumor increased tumor shrinkage by cisplatin treatment. Mechanistically, CCDC85A associated with the molecular chaperone GRP78 and GRP94, thereby inhibiting association of these negative regulators of the unfolded protein response (UPR), leading to sustained activation of PERK and downstream eIF2〈 and ATF4 upon ER stress. These data suggest a novel miR-224-3p-mediated function for CCDC85A: protection from ER stress and cisplatin resistance.

## 1 Introduction

MicroRNAs (miRNAs), single-stranded RNA molecules comprising 21–25 nucleotides, mediate a wide range of biological processes including cell proliferation, differentiation, apoptosis, and metabolism (1, 2), and play a pivotal role in communication between cancer cells and stromal cells in the tumor. For example, cancer cell-derived exosomes transform normal fibroblasts into cancer-associated fibroblasts (CAFs); in turn, CAFs secrete their own exosomes, which promote proliferation, invasion, metastasis, and epithelial-mesenchymal transition (EMT) of cancer cells via transfer of miRNAs (3–5). Exosomal miRNA-mediated crosstalk between cancer cells with stromal cells also inhibits immune responses (3, 6). Although our understanding of miRNAs and their targets in various cancer cells has been much improved, we still know comparatively little about miRNAs secreted from stromal cells via exosomes.

CAFs are the most abundant stromal cell in the tumor microenvironment of various cancers (7). In this study, we compared expression of miRNAs by normal fibroblasts (NFs) and CAFs in several patients of gastric cancer, including scirrhous (diffuse-type) gastric carcinoma, which contains abundant fibroblasts in the tumor microenvironment. We found that expression of miR-224-3p was elevated in NFs. Previous reports show that miR-224 has both protumor and antitumor functions, probably due to its context-dependent functions. For example, miR-224 has a tumor-promoting effect in nonsmall cell lung cancer (8, 9), pancreatic cancer (10), and cervical cancer (11) via targeting of homeobox D10 (HOXD10) (8, 9), androgen receptors (8, 9), thioredoxin-interacting protein (TXNIP) (8, 9), and Ras-association domain family 8 (RASSF8) (8, 9). By contrast, it has a tumor-suppressive effect on prostate cancer (12) and breast cancer (13) via targeting of tumor protein D52 (TPD52) (12) or frizzled 5 (13). MiR-224 is upregulated in gastric tumor tissues (14), where it promotes cell growth, migration, and invasion by targeting RASSF8 or RKIP (14). However, the functions of miR-224 and its target molecules in different histological types of gastric cancer are not verified, and its effects on miR-224 in tumor stromal cells are still unknown.

To investigate the effects of miR-224-3p on the tumor environment, we focused on the coiled-coil domain containing 85A (CCDC85A) as a target gene of miR-224-3p. All CCDC family members have a coiled-coil domain and play diverse roles in tumor progression (15–17). Among them, CCDC85A is a member of the delta-interacting protein A (DIPA) family, which comprises CCDC85A, CCDC85B, and CCDC85C. Each has a pair of conserved coiled-coil motifs, however, the C-terminal sequence following the coiled-coil domain shows less homology within the family. CCDC85B increases the proliferation and invasiveness of nonsmall cell lung cancer (18). Expression of CCDC85B is induced by p53, after which it regulates the activity of β-catenin through interaction with nuclear T cell factor 4 (19). By contrast, the effects of CCDC85A on the tumor environment have not been characterized.

Here, we demonstrate that CCDC85A activates cell migration and invasion, and promotes the self-guard cellular responses against endoplasmic reticulum (ER) stress. ER stress, which is induced by accumulation of unfolded proteins (UP) in the ER, is initiated by ER-located transmembrane proteins, i.e., PKR-like ER kinase (PERK), activating transcription factor 6 (ATF6), and inositol-requiring enzyme 1 (IRE1). Activated PERK phosphorylates eukaryotic initiation factor 2A (eIF2〈) and attenuates eIF2〈-mediated global translation (20). ATF6 induces chaperones, and IRE1 augments degradation of UP (20). These responses assist cell survival and alleviate ER stress. The activity of these ER stress sensors is controlled by molecular chaperones, glucose-regulated protein 78 (GRP78) and GRP94 (21, 22). GRP78 and GRP94 are HSP70-like and HSP90-like proteins, respectively, in the ER, where they play roles in the proper folding and assembly of proteins, as well as activation of transmembrane ER stress sensors. Usually, GRP78 and GRP94 bind to stress sensors in the ER to negatively regulate them. In response to ER stress, GRP78 and GRP94 dissociate from the sensors, thereby allowing activation of ER stress responses (20, 23–26).

We found that CCDC85A associated with GRP78 and GRP94, which activates PERK by interfering with the binding of GRP78 and GRP94 to PERK, leading to ER stress resistance of CCDC85A-expressing cancer cells. MiR-224-3p is a possible regulator of CCDC85A, and was expressed in NFs. NFs secreted exosomes containing miR-224-3p, which affected expression of CCDC85A by cancer cells and ameliorated tumor resistance to cisplatin. This inhibitory effects on ER-stress resistance would be lost accompanied by changing NFs to CAFs. The data suggest that miR-224 and CCDC85A are promising targets for preventing resistance of cancer cells to drugs that trigger ER stress.

## 2 Materials and methods

### 2.1 Cells

Human CAFs were obtained from the tumoral gastric wall, and NFs were obtained from the noncancerous gastric wall of the same patient (27). Briefly, the tissues were excised under aseptic conditions, and minced. The pieces were cultured in DMEM supplemented with 10% FBS, 100 IU/mL penicillin, 100 μg/mL streptomycin, and 0.5 mM sodium pyruvate, and incubated at 37°C in 5% CO_2_. The fibroblasts grew in a monolayer, and serial passage was carried out every 4–7 days. During passage, cancer cells were removed by taking advantage of the fact that fibroblasts attach to culture dishes much more quickly than cancer cells. Fibroblasts were used at passages 3–12. The purity of these NFs and CAFs was confirmed by checking expression of 〈SMA and E-cadherin (**Figure S1A**). Information about the CAF/NF-derived patients is provided in the **Supplementary Materials and Methods**. Gastric cancer cell lines HSC-43 (PRID : CVCL_A387), -57 (PRID : CVCL_A613), -59 (PRID : CVCL_A614), and -64 (PRID : CVCL_A617), and 44As3 (PRID : CVCL_XG62) and 58As9 (PRID : CVCL_XG64) cells, were isolated from patients with diffuse-type adenocarcinoma (scirrhous carcinoma); the exception was HSC-57, which was derived from intestinal-type adenocarcinoma (28–30). The human pancreatic ductal adenocarcinoma (PDAC) cell lines Capan-1 (PRID : CVCL_0237), PANC-1 (PRID : CVCL_0480), CFPAC-1 (PRID : CVCL_1119), and BXPC-3 (PRID : CVCL_0186); the glioblastoma cell lines U-87MG (PRID : CVCL_0022), T98G (PRID : CVCL_0556), and U-343MG (PRID : CVCL_S471); and the osteosarcoma cell lines HOS (PRID : CVCL_0312), SaOS-2 (PRID : CVCL_0548), and U2OS (PRID : CVCL_0042) were obtained from the American Type Culture Collection cell bank. The human osteosarcoma cell line Hu09 (PRID : CVCL_1298) and the human fetal lung normal fibroblast TIG-1-20 (PRID : CVCL_3181) were obtained from the Japanese Cancer Research Resources Bank (JCRB). Cells were cultured in DMEM containing 4,500 mg/mL glucose (U-87MG, TIG-1-20) or RPMI-1640 medium (gastric and pancreatic cancer, and osteosarcoma cell lines) containing 10% FBS. All cells were screened for mycoplasma, and the identities of the cell lines were confirmed by STR analysis. Cells were maintained in culture for less than 6 months after receipt. In some experiments, CCDC85A cDNA was stably expressed in Capan1 CCDC85A^KO^ cells by lentiviral infection, and then selected in medium containing puromycin. For viral infection, recombinant lentiviral plasmids were cotransfected along with packaging vectors into HEK293T cells (PRID : CVCL_0063) to allow the production of the viral particles. The selected cells were cloned from single colonies. The study complied with the Declaration of Helsinki and was approved by the Osaka City University Ethics Committee (approval number 2756, Osaka, Japan) and Akita University Ethics Committee (approval number a-1-3175, Akita, Japan). All patients were provided informed consent prior to the study.

### 2.2 miRNA and cDNA microarray analysis

MiRNAs were purified from NF-37 and CAF-37 using miRNeasy mini kit (Quiagen Hilden, Germany), and were subjected to GeneChip miRNA 4.0 array (Filgen, Nagoya, Japan). Total RNAs extracted from NF-37 cells and CAF-37 cells using RNeasy Mini Kit (Quiagen) were subjected to Clariom S microarray analysis (cDNA microarray; Filgen, Japan). Data were analyzed using the Microarray Data Analysis Tool Ver3.2 (Filgen) and DAVID Bioinformatics Resources 6.8 (Laboratory of Human Retrovirology and Immunoinformatics). To analyze the miRNAs differentially expressed in NF-37 or CAF-37, miRNAs were selected by the expression value which is higher than 100.0.

### 2.3 miRNA-sequencing analysis

MiRNAs were purified from NF-50 and CAF-50 using miRNeasy mini kit (Quiagen Hilden, Germany), and were subjected to microRNA sequencing analysis (Azenta life sciences, South Plainfield, NJ, USA). The microRNA difference analysis was analyzed using edgeR (V3.28.1). Genes with significant differential expression were selected according to the criteria of fold change greater than 2 and p value less than 0.05 (50 miRNAs).

### 2.4 Gene targeting of CCDC85A by CRISPR-Cas9 system

Cas9 mediated targeting of CCDC85A was performed in Capan1 cells. Human CCDC85A sgRNA sequences were chosen from predicted sequences by GeneArt CRISPR Search and design tool (Invitrogen, Waltham, MA, USA). T7 promoter and sgRNA template sequences were amplified with long liker primers by TksGflex (Takara, Shiga Japan). The purified T7-sgRNA template DNA was treated with proteinase K at 56°C for 3 hours, then purified with PCR purification kit (Takara, Shiga, Japan), and sgRNAs were transcribed using MEGA T7 transcription kit (Ambion, Austin, TX, USA). Prior to cellular genome editing, digestion of CCDC85A target genomic DNA was confirmed *in vitro* by mixing the amplified target DNA fragment, sgRNA and GeneArt Platinum Cas9 nuclease (Invitrogen, Waltham, MA USA) in restriction enzyme high buffer, and incubation at 37°C. Cas9 protein and purified sgRNA was electroporated into Capan1 cell line (1000 V, 40 ms, 2 pulses; NEPAGENE, Chiba, Japan). The single cells were cultured by limited dilution method, and each clones were selected by Western blot analysis of CCDC85A. Two independent clones were used in part of the experiments. CCDC85A^-/-^ Capan1 cells were maintained in RPMI-1640 containing 10% FBS.

T7 promoter+1(C) *target sequence* +sgRNA:

TAATACGACTCACTATAGC*ctgtccaaagtgtcggacg*GTTTTAGAGCTAGAAATAGCAAGTTAAAATAAGGCTAGTCCGTTATCAACTTGAAAAAGTGGCACCGAGTCGGTGCTTTTTTCTA

### 2.5 Stable expression of CCDC85A miRNA in U87MG cells

A system for stable expression of miRNA was generated using the BLOCK-iT Pol II miR RNAi Expression Vector Kit (Invitrogen) according to the manufacturer’s instructions. To generate the miR RNAi vectors for CCDC85A and control, the following forward primers were used:

CCDC85A miR1,

5’TGCTGAACAGCAGAGGTCCCTCAGTTGTTTTGGCCACTGACTGACAACTGAGGCCTCTGCTGTT -3’^;^

CCDC85A miR2,

5’TGCTGTCATCCAGGAAACAGCAGAGGGTTTTGGCCACTGACTGACCCTCTGCTTTCCTGGATGA -3’^;^

Control,

5’TGCTGAAATCGCTGATTTGTGTAGTCGTTTTGGCCACTGTCTGACGACTACACATCAGCGATTT -3’.

CCDC85A-miRNA expressing U87MG cells were established by transfection of miR1 or miR2 above into the parent cells, and selected in DMEM containing 4,500 mg/mL glucose and blasticidin (Invitrogen) at a concentration of 10 μg/mL for 3 weeks. The selected cells were collected and used in bulk (referred to miR1 cells and miR2 cells) for experiments.

### 2.6 Preparation of fibroblasts-derived exosomes

Normal stomach fibroblasts (NFs) were cultured under hypoxia (O_2_, 1.0%) using a CO_2_-multigas incubator (APM-30D, ASTEC Fukuoka, Japan) for 40 h in medium lacking FBS. The medium was collected, and exosomes were purified from the supernatants by ultracentrifugation as described previously (31). Briefly, culture supernatants were cleared of cell debris by centrifugation at 300 × g, and centrifuged at 2,000 × g for 20 min to pellet large vesicles and apoptotic vesicles. The supernatant was centrifuged at 10,000× g for 30 min to remove microvesicles, and then centrifuged again at 100,000 × g for 2 h to pellet exosomes.

### 2.7 RT-PCR and TaqMan micro RNA assays

The expression of miRNAs was initially examined by detection of each miRNA precursor using Simple miRNA detection kit (BioDynamics Lab Inc. Tokyo, Japan). Briefly, miRNAs were ligated to a universal oligonucleotide tag, and tagged miRNAs were reverse-transcribed. The resulting cDNA was amplified with a miRNA specific forward primer and a universal reverse primer corresponding to the tag sequence. The PCR products were examined by polyacrylamide gel electrophoresis. Specific forward primers of representative miRNAs are as follows:

miR-224-Fw: 5’- TCAAGTCACTAGTGGTTCC-3’,

miR-383-Fw: 5’- CTCAGATCAGAAGGTGATT-3’,

miR-497-Fw: 5’- ACCCCGGTCCTGCTCC-3’,

miR-195-Fw: 5’- AGCTTCCCTGGCTCTAGCA-3’,

miR-708-Fw: 5’- TGCCCTCAAGGAGCTTACA-3’,

miR-137-Fw: 5’- GGTCCTCTGACTCTCTTCG-3’,

miR-218-Fw: 5’- AGCGAGATTTTCTGTTGTG-3’,

miR-10-Fw: 5’- TGTCTGTCTTCTGTATATA-3’,

miR-34-Fw: 5’- AGTTACTAGGCAGTGTAGT-3’,

miR-145-Fw: 5’- ACCTTGTCCTCACGGTCCA-3’,

The expression of human miR-224-3p was quantified using TaqMan microRNA assays using U47 as an internal control. Total RNAs were reverse transcribed to cDNAs using miRNAs specific primers using TaqMan MicroRNA Reverse Transcriptase kit (4366596, Applied Biosystem). In brief, 10 ng of total RNA was mixed with dNTPs, reverse transcription buffer, RNase inhibitor, and miRNA specific primer (4427975) and reverse transcribed to a final reaction mixture of 15 μl. Thereafter, the reaction mixture was subjected to thermal cycle at 16°C for 30 min, 42°C for 30 min, and 85°C for 5 min. The PCR was then run at 95°C for 10 min, and for 40 cycles at 95°C for 15 s and 60°C for 1 min. Samples were run in triplicate.

### 2.8 Luciferase reporter assay

The 3′UTR of CCDC85A mRNA containing miR-224 binding sites (1-1,487) was PCR-amplified and inserted into down-stream of a fire fly luciferase reporter gene in the pGL4.5 vector containing CMV promoter (Promega). Renilla luciferase reporter gene was used as a control. 293T cells were transfected with the luciferase reporter constructs together with or without miR-224-3p mimics or the control mimics using Lipofectamine 3000 transfection reagent. At 24 h after transfection, cell lysates were collected and luciferase intensity was determined using the Dual-Luciferase Reporter Assay System (Promega) according to the manufacturer’s instructions. Luciferase activity was measured by the luminometer (GL-200, Microtec Chiba, Japan). The raw values of relative light unit (firefly)/relative light unit (Renilla) (F/R ratio) were calculated.

### 2.9 Flow cytometric analysis

**Cell cycle assay:** Cell cycle assay was performed by quantitation of DNA content. Briefly, cells were fixed in cold 70% ethanol at 4°C for 2 h, rinsed by PBS twice, and treated by RNase (0.25 μg/mL) at 37°C for 30 min. Cells were then labeled by Propidium Iodide (BD biosciences), and subjected to FACS analysis using a BD FACSAriaTM III (BD Biosciences) with FACSDiva and BD FlowJo software (BD biosciences).

**Apoptosis assay:** Cells were labeled by 7-amino-actinomycin D (7-AAD) (Miltenyi Biotec) to identify dead cells, and subjected to FACS analysis as described above.

### 2.10 Immunofluorescence staining

Cells were fixed with 4% paraformaldehyde in PBS and permeabilized for 5 min with 0.1% Triton X-100. Cells were pre-incubated in 3% bovine serum albumin for 30 min and incubated with specific primary antibodies (1:500 dilution) for 1 h at room temperature. After washing, cells were incubated with Alexa Fluor-conjugated secondary antibodies (Invitrogen) for 1 h at room temperature. Images were obtained using an LSM780 or LSM980 (Zeiss) confocal microscope and processed using Zen software (Zeiss).

### 2.11 Patients and tissue samples

Patients with gastric cancer or pancreatic ductal adenocarcinoma who underwent surgery at Akita University from April 2007 to March 2017, for whom sufficient clinical information was available and accurate prognostic follow-up was possible, were included in the study. The study was approved by the Akita University Ethics Committee (approval number 1662, 2190 Akita, Japan). All specimens were handled and made anonymous according to the ethical and legal standards.

### 2.12 *In vivo* tumor transplantation

Specific pathogen-free (SPF) BALB/c^nu/nu^ mice (6-week-old, males) were obtained from CLEA Japan, Inc. (Tokyo, Japan). The mice were bred under specific pathogen-free conditions at the Animal Research Laboratory Bioscience Education-Research Center of Akita University.

All animal experimental protocols were approved by the Committee for Ethics of Animal Experimentation (approval number a-1-3175, Akita, Japan), and the experiments were conducted in accordance with the guidelines for Animal Experiments at Akita University. Fluorescence-labeled cancer cells (1 × 10^6^ cells each) suspended in 150 μL of medium were injected subcutaneously into 6-week-old nude mice. In some experiments, cisplatin (CDDP; 2.5 mg/kg body weight) was injected intraperitoneally once every other day (4–6 times in total, as indicated). Tumor size (diameter) was measured every day using a caliper. On the indicated days, mice were sacrificed by cervical dislocation, and subcutaneous tumors were resected. Five mice were used per group, and randomization was not performed. When assessing the outcome, the investigators were blinded to the group allocation.

### 2.13 Statistical analysis

Data are expressed as the mean ± standard deviation. Statistical significance was calculated using the Student’s t-test. P values <0.05 were considered statistically significant.

Other methods are described in the **Supplementary Materials and Methods**.

## 3 Results

### 3.1 Downregulation of miR-224-3p in tumor fibroblasts affects cell migration and proliferation

**Figure 1:**
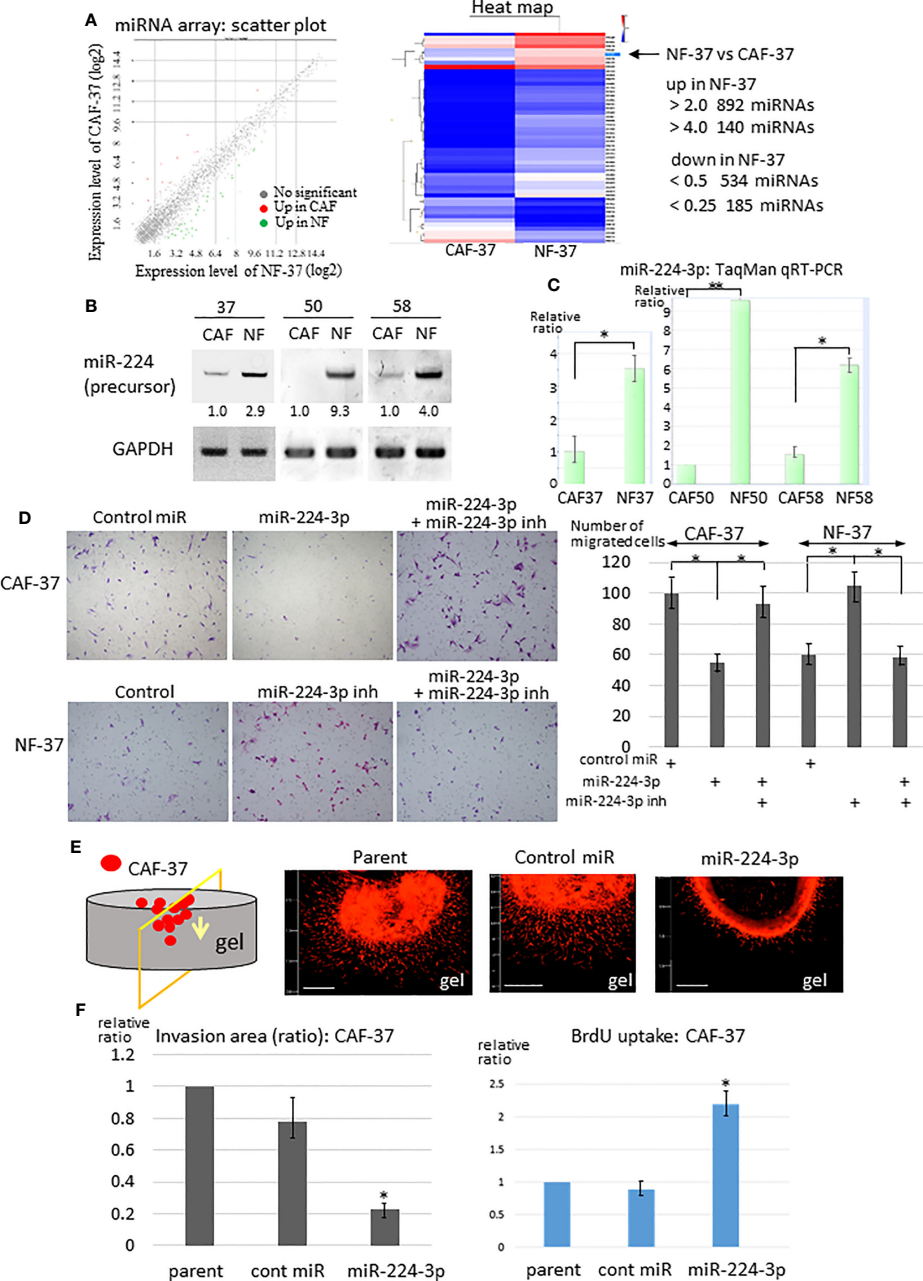
Downregulation of miR-224-3p in CAFs affects cell proliferation, migration, and invasion. **(A)** RNA was purified from NF-37 and CAF-37 cells, and subjected to miRNA microarray analysis. Left: Scatter plot of the genes in CAF/NF. The green and magenta dots represent miRNAs that are up- or downregulated, respectively, in CAF-37 cells. Right: Heat map showing the comparison between CAF-37 and NF-37. **(B)** RT-PCR of miR-224 precursor was performed on CAFs and NFs isolated from three patients. Amplified products were analyzed by electrophoresis on polyacrylamide gels. GAPDH was used as the internal control. **(C)** Validations of miR-224-3p by qRT-PCR using TaqMan microRNA assays. Results are expressed as the relative ratio to CAFs in each. *P < 0.05, **P < 0.01. **(D)** Transwell assay was performed by CAF-37 and NF-37 with transfection of control or miR-224-3p miRIDIAN, or miR-224-3p miRIDIAN with miR-224-inhibitor. Cells were seeded onto a Transwell membrane, and harvested at 12h and cells that migrated to the bottom surface of the membrane were counted. Representative fields from each experiment are shown (results represent three independent experiments, each in duplicate). *P < 0.01. **(E)** 3D gel invasion assay of CAF-37 transfected with the control or miR-224-3p miRIDIAN. DiI-labeled cells were plated on a gel, and the images were taken after 5 days. Bar, 500 μm (left bottom in each panel). **(F)** The invasion area was measured (the method and examples are described in the **Supplementary Materials and Methods** and **Figure S2A**, respectively), and is expressed as the relative ratio to control untreated parent cells. *P < 0.01. **(G)** Cell proliferation was determined by BrdU uptake of CAF-37 cells. The results from three independent experiments are shown as means +/- SD. *P < 0.05 by Student’s *t*-test.

To examine the difference in the miRNA profiles of NFs and CAFs in scirrhous gastric carcinoma, miRNAs were isolated from NF-37 and CAF-37 cells (32) and examined by microarray analysis. We detected 140 upregulated miRNAs (>4-fold) and 185 downregulated miRNAs (<0.25-fold) in NF-37 compared with CAF-37 (**Figure 1A**, **S1B**, left) cells, and the greatest increase or decrease in CAFs were shown in **Figure S1C**. Among them, we focused on miRNAs upregulated in NFs (>5-fold), because their targets may include pro-tumor, therapeutic target molecules (**Figure S1B** left, **S1C**). To further validate by RT-PCR, miRNAs were isolated from NFs and CAFs from three different patients of gastric cancer. At first, we evaluated the amount of each precursor miRNA; we selected miR-224 because it was present at significantly higher amounts in NF cells (not only in NF/CAF-37 but also in NF/CAF-50 and NF/CAF-58) (**Figure 1B**). Consistent with this, miR-224 was also detected as a significantly upregulated miRNA in NF-50 compared with CAF-50 by microRNA-sequencing analysis (**Figure S1B**, right). Expression of other precursor miRNAs was elevated in NFs from only one or two patients, or not reproducibly upregulated in NF-37 (**Figure S1D**). QRT-PCR confirmed that miR-224-3p was downregulated in CAFs from the three patients (**Figure 1C**).

Because exaggerated invasion of CAFs guides cancer cells and promotes tumor expansion (33), we examined the effects of miR-224-3p on migration and invasion of fibroblasts. A miR-224-3p mimic (miRIDIAN miR-224-3p) was introduced into CAF-37 cells to determine whether it altered migration and invasion. MiR-224-3p decreased migration of CAF-37 significantly in a Transwell assay (**Figure 1D**, upper panel). By contrast, the inhibitor of miR-224-3p promoted migration of NF-37 cells (**Figure 1D**, bottom panel). In addition, a 3D gel invasion assay of CAFs revealed that although miR-224-3p mimic decreased invasion by CAF-37 cells (**Figures 1E, F**, **Figure S2A** top), it increased proliferation, as determined by measuring BrdU uptake (**Figure 1G**).

### 3.2 CCDC85A is a novel target of miR-224-3p and promotes invasion of fibroblasts

**Figure 2:**
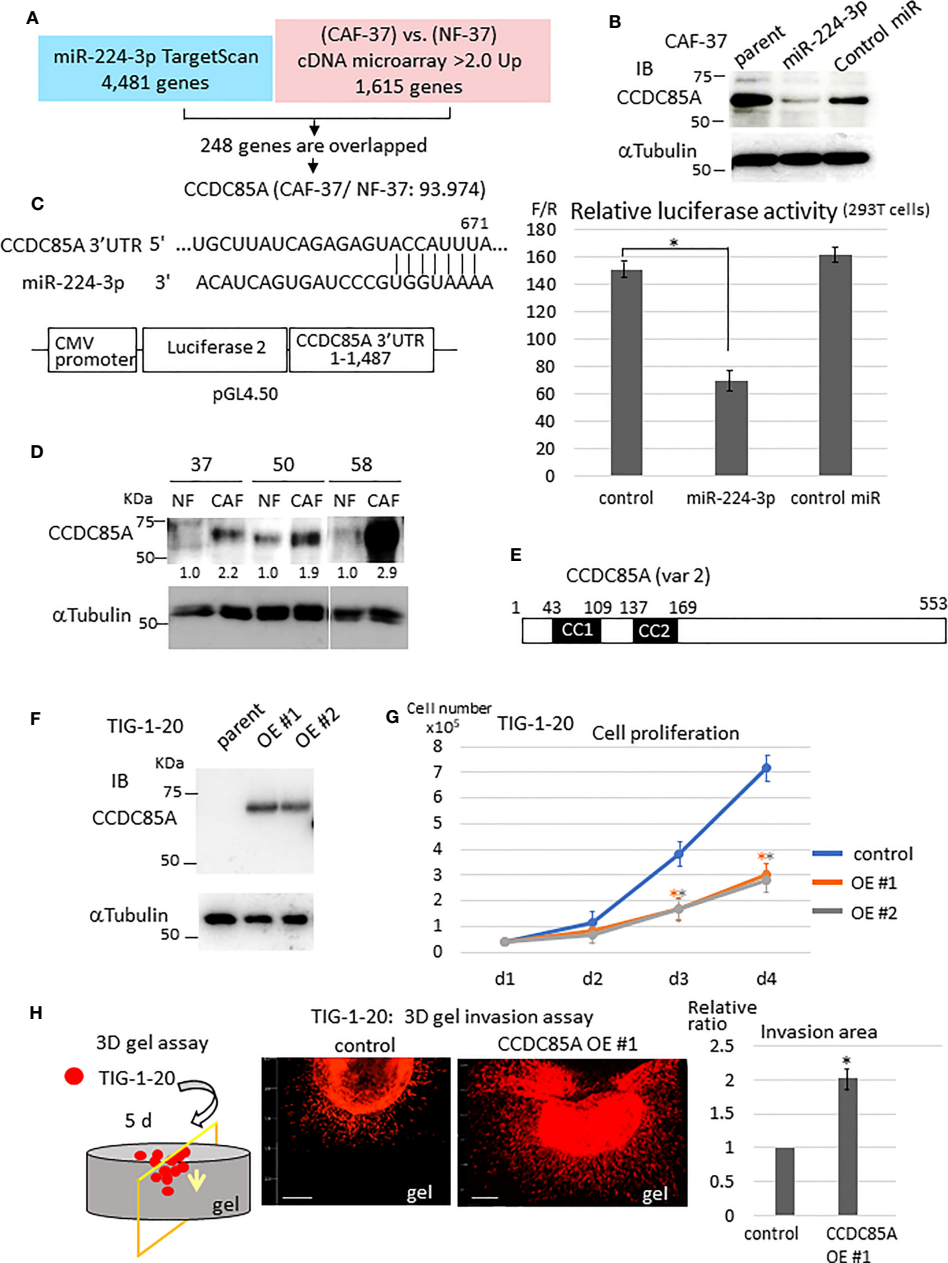
CCDC85A is a novel target of miR-224-3p, which promotes invasion of fibroblasts. **(A)** Target genes of miR-224-3p predicted by TargetScan software were checked against genes that were upregulated in CAF-37 compared with NF-37. Two hundred and forty-eight overlapped genes (upregulated in CAF-37 by more than 2-fold compared with NF) were identified as candidate miR-24-3p targets. Among them, CCDC85A, the most elevated gene in CAF (93.974-fold) was selected. **(B)** CAF-37 cells were transfected with miR-224-3p or control miRIDIAN or left untreated. Cells were lysed at Day 3 and subjected for western blot analysis with an anti-CCDC85A antibody. **(C)** Top: The predicted binding sequences of miR-224-3p in the 3’UTR of CCDC85A. Bottom: A schematic diagram showing construction of the reporter plasmid. Right: Dual luciferase reporter assay (performed as described in Materials and methods). Relative luciferase activity (F/R, Fire fly: sample/Renilla: control) is shown. Control cells were not transfected with miRNA. The results from three independent experiments are shown as means +/- SD. *P < 0.01. **(D)** Expression of CCDC85A by NF and CAF derived from three scirrhous gastric cancer patients was examined by western blotting. Intensity of each band was quantified, and expression of CCDC85A was normalized by α−Tubulin, and expressed as the relative ratio to NF. **(E)** Structure of human CCDC85A. cc1, cc2: coiled-coil domains. Amino acids are numbered. **(F)** Western blot analysis of CCDC85A in TIG-1-20 cells stably expressing CCDC85A variant 2 (two clones; OE #1, OE #2). **(G)** *In vitro* proliferation of TIG-1-20 cells overexpressing CCDC85A variant 2 (two clones; OE #1, OE #2) was evaluated by counting the cells under standard culture conditions. Data points indicate the average results from three dishes. *P < 0.01. **(H)** 3D gel invasion assay of TIG-1-20 transfected with CCDC85A var 2. DiI-labeled cells were plated on a gel, and images were obtained after 5 days (see **Supplementary Materials and Methods**). The invasion area is expressed as the relative ratio to control untreated parent cells. *P < 0.01. Bar, 500 μm. The different color asterisk indicate the significance of OE1 (orange) or OE2 (gray) compared with the control.

Next, we examined the target genes of miR-224-3p in NFs. For this purpose, target genes predicted by TargetScan Human 8.0 (34) were checked against the list of genes differentially expressed by CAF-37 and NF-37 cells (determined by cDNA microarray analysis) (33) (**Figure 2A**). Among 4,481 candidate targets of miR-224-3p, 248 that were upregulated in CAF-37 relative to NF-37 (ratio >2.0) were selected. Among them, *ccdc85A*, which showed the highest relative increase in CAF/NF-37 cells, was analyzed further (**Figure S1E**). We performed a similar analysis using miRmap to detect *ccdc85A* as a predicted miR-224-3p target (35) (**Figure S1F**).

Expression of CCDC85A in CAF-37 cells was greatly reduced by introduction of miRIDIAN miR-224-3p (**Figure 2B**). Also, the CCDC85A 3’UTR contained the sequence targeted by miR-224-3p (**Figure 2C**, left upper), and the gene reporter assay revealed that miR-224-3p reduced the luciferase activity regulated by the CCDC85A 3’UTR (**Figure 2C**). When we compared expression of CCDC85A in NF and CAF, we found that it was elevated in CAFs in which miR-224-3p was downregulated (**Figure 2D**).

Next, we examined the effects of CCDC85A-overexpressing fibroblasts. To this end, CCDC85A full-length cDNA (accession: NM_001080433, variant 2) was introduced into human normal fetal fibroblasts (TIG-1-20) (**Figures 2E, F**); this was done because the cDNA corresponding to CCDC85A variant 2 was frequently amplified by RT-PCR of CAF-37 mRNA (data not shown). In TIG-1-20 cells, miR-224-3p was low level (**Figure S2B**, left), which was not influenced by overexpression of CCDC85A (**Figure S2B**, right). Elevated expression of CCDC85A attenuated proliferation of TIG-1-20 cells (**Figure 2G**), and did not affect cell transformation (**Figure S2C**). By contrast, CCDC85A increased the invasiveness of TIG-1-20 cells (**Figure 2H**, **Figure S2A**, bottom).

### 3.3 Elevated expression of CCDC85A upregulates cancer cell migration and invasion

**Figure 3:**
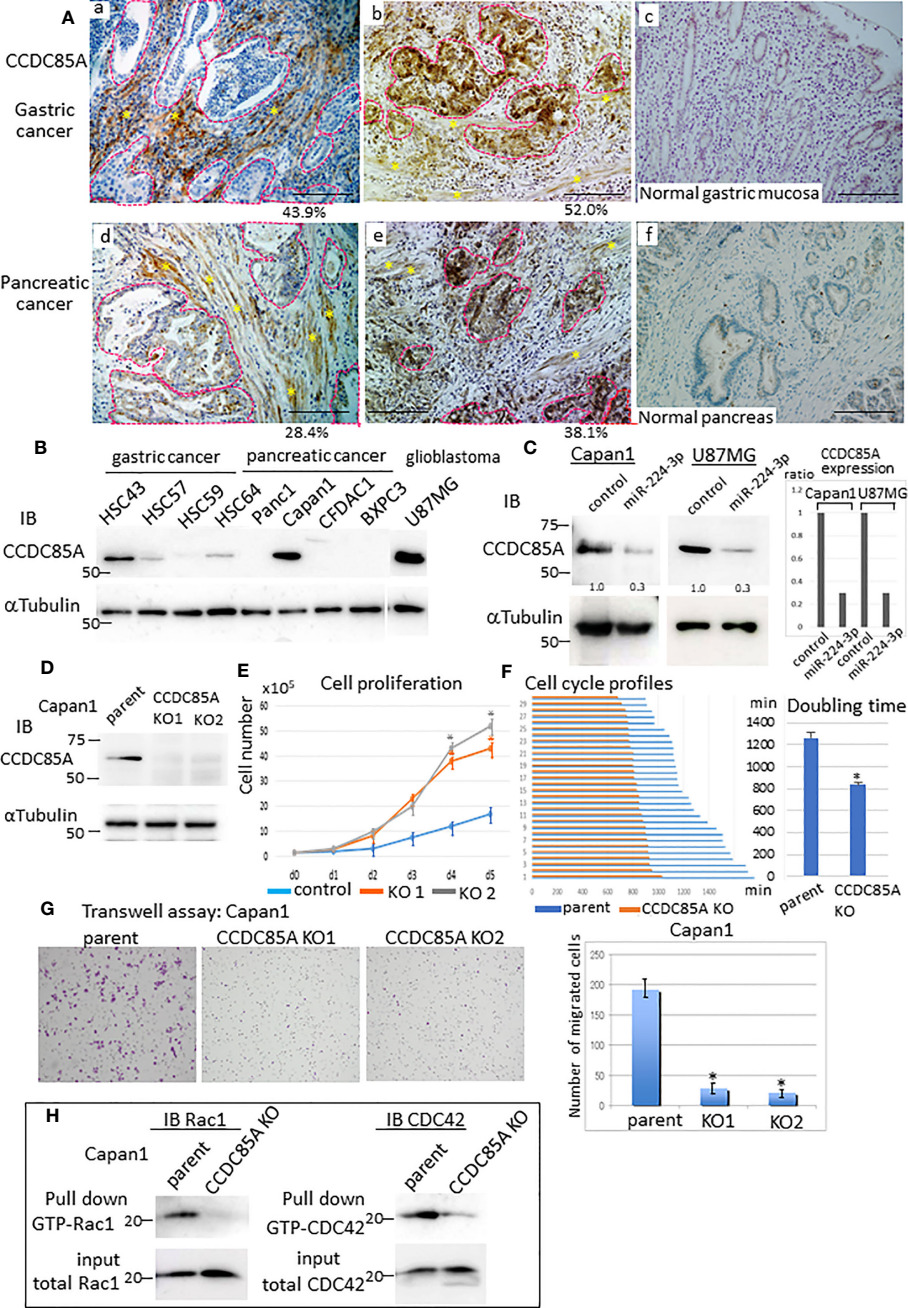
Increased expression of CCDC85A in tumors promotes cancer cell migration and invasion. **(A)** Immunohistochemical analysis of CCDC85A in human gastric cancer (top panels) and pancreatic cancer specimens (bottom panels). Representative cancer cell nests are demarcated by dotted lines (red), and CAF regions are marked by asterisks. Normal regions (c, f) are shown to the right of each image. Representative images are shown. Bar, 100 μm. Percentage of CCDC85A positive area/total area in each specimen was shown in the bottom. Example of the quantification of each area was shown in **Figure S2D**. **(B)** Expression of CCDC85A by cancer cells was examined by western blot analysis. **(C)** Capan1 and U87MG cells were transfected with the control or miR-224-3p miRIDIAN and subjected to western blot analysis to detect expression of CCDC85A. Right: Intensity of each band was quantified, and expression of CCDC85A was normalized by 〈−Tubulin, and expressed as the relative ratio to the control miRNA transfected cells. **(D)** Capan1 parent cells or CCDC85A knockout cells were examined for CCDC85A expression by western blotting. KO1 and KO2 indicate two independent clones. **(E)** Proliferation of Capan1 parent cells (control) or CCDC85A^KO^ cells was evaluated by counting the cells under standard culture conditions. Data points indicate the average results from three dishes. *P < 0.01. **(F)** Cells were time-lapse monitored to evaluate the cell cycle morphologically. Bar graph indicates the cell cycle (duration (min) of M phase to M phase) of 30 individual cells of each control and CCDC85A^KO^. (Right) Graph indicates the average results of each 30 individual cells. *P < 0.01. **(G)** Transwell assay of Capan1 parent cells or CCDC85A knockout cells. The number of migrated cells (shown on the right) was counted in three independent experiments. *P < 0.01. **(H)** Cell lysates were prepared from cancer cells, pulled-down by GST-PBD, and then immunoblotted with anti-Rac1 or anti-CDC42 to detect GTP-bound Rac1 or CDC42 (activated). Expression of total Rac1 or CDC42 in the input is shown at the bottom. a, b, d, e are individual different patients.

To confirm CCDC85A expression in tumors, we performed immunohistochemical analysis of several cases of gastric and pancreatic cancer. CCDC85A was detected in both CAFs and cancer cells to varying degrees (**Figures 3Aa, b, d, e**, **S2D**), but rarely in normal gastric mucosa or in normal pancreas secretory ducts (**Figures 3Ac, f**). Because CCDC85A was expressed not only in CAFs but also in some cancer cells (**Figure S3A**), we examined CCDC85A expression in various human cancer cell lines. High expression of CCDC85A was detected in HSC43 (scirrhous gastric cancer), Capan1 (pancreatic ductal adenocarcinoma), and U87MG (glioblastoma) (**Figure 3B**) cells. Suppression of CCDC85A expression by miRIDIAN miR-224-3p was observed in Capan1 and U87MG cells (**Figure 3C**), supporting the notion that CCDC85A is the target of miR-224-3p.

To examine the effect of CCDC85A on cancer cells, we targeted the CCDC85A gene in Capan1 cells (**Figure 3D**). First, we assessed whether CCDC85A affects the growth of these cells *in vitro*. Depletion of CCDC85A from Capan1 cells increased cell growth (**Figure 3E**). We then examined the cell cycle. Cultured Capan1 cells were monitored by time-lapse photography, and the cell cycle (duration of M phase to M phase) of 30 randomly selected cells was measured. The data show that CCDC85A^KO^ cells went through the cell cycle faster than parental cells (**Figure 3F**). When we examined the cell cycle by flow cytometry, the percentage of CCDC85A^KO^ cells in the G1 phase was slightly reduced, and S phase was higher than that of parental cells (**Figure S3B**).

Next, a Transwell assay was performed to assess migration and invasion of Capan1 cells. The results showed that the number of migrated or invaded CCDC85A^KO^ cells was clearly lower than that of the parent cells (**Figure 3G**, **Figure S3C**). We then examined activation of Rac1 and CDC42, which are small GTPases that promote cell migration via formation of lamellipodia and filopodia (36). To detect activated Rac1 and CDC42, pulldown assays were performed using the GTP-Rac1/CDC42-binding domain of PAK1 as a probe (**Supplementary Materials and Methods**). Knockout of CCDC85A in Capan1 cells reduced activation of Rac1 and CDC42 (**Figure 3H**). In addition, depletion of CCDC85A altered the morphology of Capan1 cells, particularly under hypoxic conditions. We observed that hypoxic control cells were elongated and scattered, suggesting EMT, whereas EMT-like changes were rather mild in CCDC85A^KO^ cells (**Figure S3D**). Consistent with this, elevation of EMT-related molecules (HIF1〈, Slug, and N-cadherin) in CCDC85A^KO^ cells was attenuated, while that of E-cadherin was upregulated (**Figure S3E**). Moreover, the expression of TGFβ was elevated in control parental cells under hypoxic conditions, whereas the expression was attenuated in CCDC85A^KO^ cells (**Figure S3F**).

Similar results were obtained using U87MG cells. CCDC85A in U87MG cells was downregulated by stable transduction of CCDC85A miRNA (**Figure S4A**). When U87MG cells were plated on a gel, control cells contracted the gel surface and collected at the center of the gel. This gel contractile property was weak in CCDC85A-miR cells (**Figure S4B**, upper panel). Consistent with this, downregulation of CCDC85A greatly reduced invasion of the gel by U87MG cells (**Figure S4B**, bottom), while proliferation of CCDC85A miR cells was again increased (**Figure S4C**).

### 3.4 Exosomes secreted from fibroblasts contain miR-224-3p, which reduces expression of CCDC85A by recipient cancer cells

MiRNAs are incorporated in exosomes and transferred to surrounding cells. Therefore, we asked whether miR-224-3p was contained in exosomes secreted from NFs, and whether it reduced expression of CCDC85A by recipient cells. When miRNAs were extracted from exosomes purified from the culture medium of NF-37 or CAF-37 cells grown under hypoxic conditions, more miR-224-3p was detected in exosomes from NFs (**Figure S4D**). Treatment of Capan1 cells with NF-secreted exosomes (NF-exo) revealed that expression of CCDC85A in Capan1 cells decreased; however, this was prevented by treatment with a miR-224-3p inhibitor (**Figure S4E**). Accordingly, migration of Capan1 cells was suppressed by NF-exo, whereas this was prevented by transduction of the miR-224-3p inhibitor (**Figure S4F**). By contrast, transduction of the miR-224-3p inhibitor alone did not have a marked effect on expression of CCDC85A in Capan1 cells (**Figure S4E**) or U87MG cells (data not shown). These results suggest that CCDC85A expression in cancer cells may be, at least partially, modified by surrounding fibroblasts.

### 3.5 CCDC85A alleviates ER stress and contributes to resistance to cisplatin

**Figure 4:**
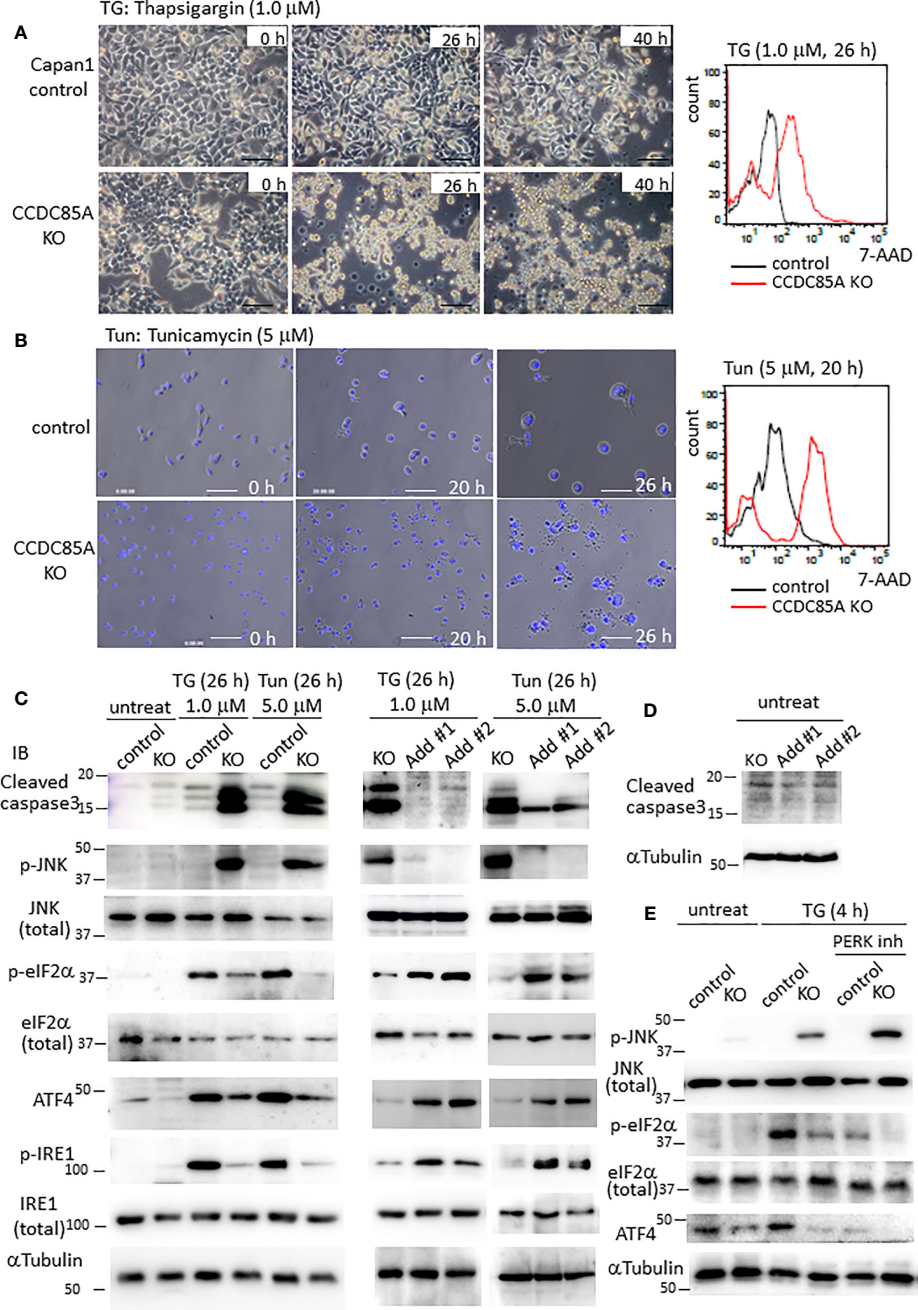
CCDC85A alleviates ER stress and contributes to resistance against cisplatin treatment. **(A, B)** Appearance of Capan1 control (top panels) and CCDC85A^KO^ cells (bottom panels) exposed to thapsigargin (TG) **(A)** or tunicamycin (Tun) **(B)** as indicated. Bar, 100 μm. **(B)** Cells were labeled by Hoechst 33342, and nuclear fragmentation was monitored to evaluate apoptosis. Bar, 100 μm. Right panels: Control and CCDC85A^KO^ Capan1 cells were subjected to flow cytometry analysis to investigate apoptosis. Histogram of 7-AAD was shown. **(C, D)** Capan1 control, CCDC85A^KO^, and CCDC85A addback cells were treated with TG or Tun as indicated **(C)** or left untreated **(D)**. Cell lysates were prepared and subjected to western blot analysis. The antibody specific for p-eIF2〈 detects phosphorylation on Ser51, and that specific for p-IRE1 detects phosphorylation on Ser724. **(E)** Capan1 CCDC85A^KO^ cells and control cells were treated as above or pretreated with PERK inhibitor I (5 μM) for 30 min prior to addition of TG. Cell lysates were prepared 4 h after TG treatment and subjected for western blot analysis.

Resistance of cancer cells to chemotherapeutic agents such as cisplatin is a major problem, and this phenomenon is affected by cellular responses to ER stress. Treatment with ER stress activators thapsigargin (TG) or tunicamycin (Tun) triggered apoptosis in Capan1 CCDC85A^KO^ cells to a greater extent than in control cells (**Figures 4A, B**). Consistent with this, TG and Tun induced expression of cleaved caspase-3 and activation (phosphorylation) of JNK in Capan1^KO^ cells; however, this was far less evident in Capan1 control cells under the same conditions (**Figure 4C**; left panel).

PERK and IRE1 are ER-located sensor proteins that are activated in response to ER stress. Activated PERK prevents accumulation of UP by phosphorylates eIF2〈, and augments autophagy by upregulation of ATF4; both facilitate cell survival (37, 38). Phosphorylation of eIF2〈 and induction of ATF4 induced by TG or Tun were attenuated in Capan1 CCDC85A^KO^ cells (**Figure 4C**, left). In addition, phosphorylation (activation) of IRE1 was alleviated in Capan1 CCDC85A^KO^ cells (**Figure 4C**, left). By contrast, these events were prevented by addback of CCDC85A (**Figure 4C**, middle and right panels). In the absence of TG or Tun, there was no apparent apoptosis in any of these cells (**Figure 4D**). In addition, pretreatment of cancer cells with a PERK inhibitor augmented TG-induced apoptosis, particularly in CCDC85A^KO^ cells (**Figure S4G**), accompanied by JNK activation and attenuation of eIF2〈 phosphorylation and ATF4 expression (**Figure 4E**, right). These data suggest that CCDC85A promotes PERK-mediated cell survival responses during stress conditions.

Similar results were obtained for U87MG glioblastoma cells. Treatment of CCDC85A-attenuated U87MG cells with TG or Tun led to severe apoptosis, accompanied by induction of cleaved casapase-3 (**Figure S5A, B**). Again, phosphorylation of eIF2〈 and IRE1 was attenuated in U87MG CCDC85A miR cells (**Figure S5B**). Coincident with this, PERK activation, evaluated by measuring PERK phosphorylation on Thr 982, was also attenuated in CCDC85A miR cells (**Figure S5B**). Because cisplatin (cis-diamminedichloro-platinum: CDDP) also stimulates ER stress in cancer cells (indeed, it is often used for chemotherapy of gastric and pancreatic cancers, and for glioblastoma), we evaluated the protective effects of CCDC85A on CDDP-treated Capan1 and U87MG cells. As expected, CDDP-induced apoptosis, accompanied by increased levels of cleaved caspase-3 and phosphorylated JNK, while phosphorylation of eIF2〈 and IRE1 was decreased by downregulation of CCDC85A (**Figure S5C**).

**Figure 5:**
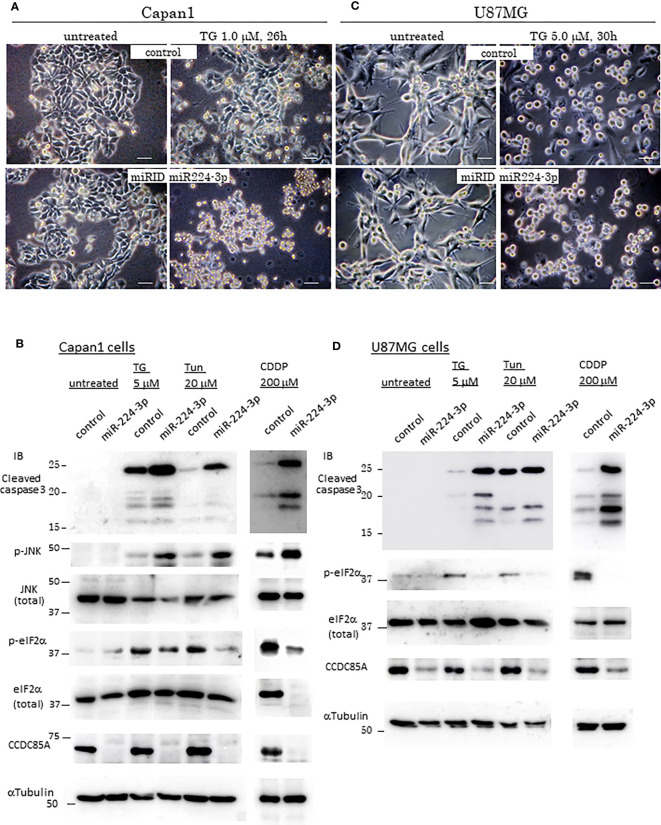
MiR-224-3p aggravates ER-stress/Cisplatin induced apoptosis. **(A, C)** Appearance of control cells of Capan1 or U87MG (top panels), and miR-224-3p mimic (miRIDIAN) transfected cells (bottom panels) after exposure to thapsigargin (TG) as indicated above the panels. Representative images are shown. Bar, 40 μm. **(B, D)** Capan1 or U87MG cells (control or miRIDIAN miR-224-3p transfected) were treated by TG, Tun or CDDP, or left untreated for 30h. Protein lysates were prepared and subjected for Western blot analysis with indicated antibodies.

Next, we examined the effects of miR-224-3p on ER stress in cancer cells. Introduction of miRIDIAN miR224-3p into Capan1 cells (**Figures 5A, B**) and U87MG cells (**Figures 5C, D**) increased apoptosis and cleaved caspase-3 levels while suppressing eIF2〈 phosphorylation in response to TG, Tun, or CDDP in both cancer cells. In addition, introduction of miR224-3p in CAF50 cells also showed the similar results (**Figure S6A**). Taken together, these results suggest that CCDC85A alleviates ER stress, which is (at least in part) dependent on activation of UPR-mediated cell survival. In this regard, we further examined expression of some inflammation-related cytokines because CCDC85A promotes activation of IRE-1, which is known to induce proinflammatory cytokines (39, 40). Treatment of Capan1 cells or U87MG cells with TG induced production of TNF〈 and IL-6, and activated STAT3, which were attenuated by downregulation of CCDC85A (**Figure S6B**). Considering that the IL-6/STAT3 pathway promotes cell growth and survival (41), it may also contribute to CCDC85A-mediated ER stress resistance.

### 3.6 CCDC85A regulates ER stress responses by interfering with the association between PERK and GRP78/GRP94

**Figure 6:**
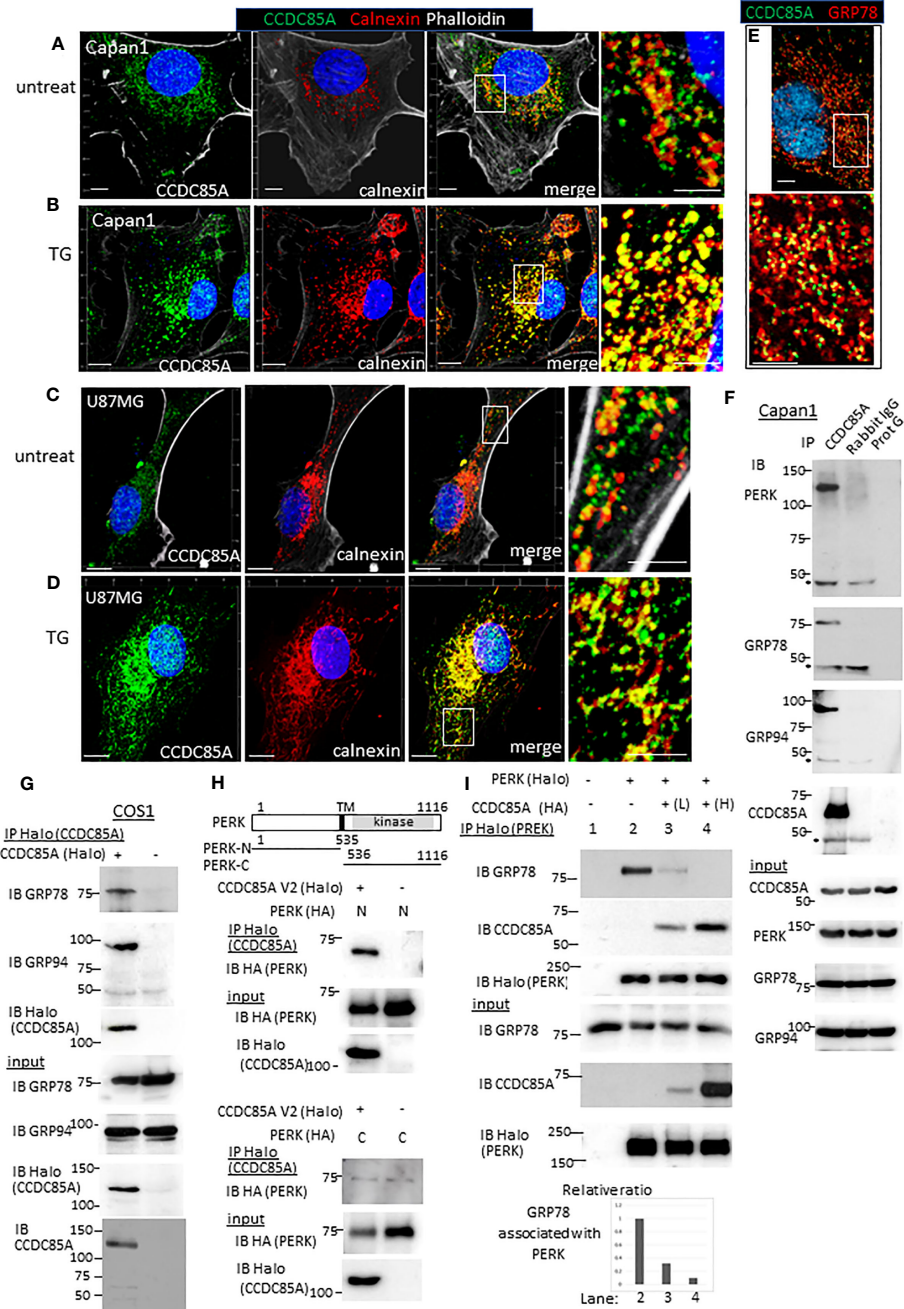
CCDC85A localizes in the ER and disrupts the interaction between PERK and GRP78/GRP94. **(A–D)** Capan1 **(A, B)** or U87MG cells **(C, D)** were treated with TG (B, 1 μM to Capan1; D, 5 μM to U87MG) for 24 h or left untreated. Cells were fixed and immunostained with anti-CCDC85A (green), anti-calnexin (red) antibodies, and phalloidin (white) as indicated above the panel. Bar, 10 μm. The boxed areas are enlarged to the right of each panel (Bar, 5 μm). **(E)** Capan1 cells were immunostained with anti-CCDC85A (green) and anti-GRP78 (red) antibodies. **(F)** Cell lysates of Capan1 were immunoprecipitated by an anti-CCDC85A antibody or rabbit IgG control antibody, or protein-G agarose alone and coprecipitated PERK, GRP78 or GRP94 was detected by each antibody. Asterisk indicates the heavy chain of IgG. **(G)** COS1 cells were transiently transfected with the plasmids encoding CCDC85A tagged with Halo at C-terminus. Cell lysates were prepared 30 h after transfection and subjected to immunoprecipitation (IP) of CCDC85A using Halo-tag magnetic beads. Coprecipitated endogenous GRP78 or GRP94 was detected by each antibody. Expression of transfected genes in cell lysates (input) is shown in the bottom panels. **(H)** Protein lysates of COS1 cells transfected with C-terminally HA-tagged PERK deletion mutant forms (illustrated at the top) and CCDC85A-Halo were subjected for IP to detect CCDC85A; coprecipitated PERK deletion forms were detected by an anti-HA antibody. Weak bands observed in the bottom panel indicate the background level of coprecipitated PERK-C. **(I)** COS1 cells were transfected with the plasmids indicated above the lanes. Lanes 3 and 4 contain lysates of cells transfected with increasing amounts of CCDC85A tagged with HA. PERK was immunoprecipitated by Halo magnetic beads, and coprecipitated endogenous GRP78 and transfected CCDC85A were detected by anti-PERK or anti-CCDC85A antibody. [**(I)**, bottom] The intensity of the bands representing coprecipitated GRP78 was measured and normalized to the intensity of input GRP78. The results are expressed as a relative ratio.

Next, we examined intracellular localization of CCDC85A. Because CCDC85A contributes to ER stress resistance, we examined its localization by coimmunostaining with calnexin, a marker of the ER. In untreated Capan1 and U87MG cells, CCDC85A showed a granular staining pattern, and partially localized with calnexin-positive ER structures (**Figures 6A, C**). After these cells were treated with TG, CCDC85A accumulated in the ER, and colocalization with calnexin was more evident (**Figures 6B, D**). CCDC85A also colocalized, at least partially, with the molecular chaperone GRP78 (**Figure 6E**).

Next, we examined the association between CCDC85A and molecules involved in ER stress responses. Activation of ER-localized transmembrane proteins PERK, IRE-1, and ATF6 are regulated by molecular chaperones GRP78 and GRP94. Immunoprecipitation analysis revealed that CCDC85A formed a complex with PERK, GRP78 and GRP94 (**Figure 6F**). In addition, the association of endogenous CCDC85A with GRP78 or GRP94 was augmented by treatment with thapsigargin (**Figure S7A, B**). In COS1 cells, exogenously transfected CCDC85A associated with GRP78, GRP94 (**Figure 6G**) and PERK (**Figure S7C**). When we examined the amino-terminus (ER lumen) and carboxyl-terminus (cytosol) of PERK, we found that CCDC85A associated with the N-terminal region, suggesting that CCDC85A interacts with PERK in the ER lumen (**Figure 6H**).

In response to accumulation of UP in the ER, GRP78 dissociates from PERK, leading to dimerization and activation of PERK. Overexpression of CCDC85A interfered with the interaction between PERK and GRP78 or GRP94 (**Figure 6I**, **S7D**). Consistent with this, knockdown of CCDC85A augmented the association of endogenous PERK with GRP94 (**Figure S7E**). These results suggest that CCDC85A activates PERK-mediated UPR by interfering with binding of GRP78 or GRP94 to PERK, thereby alleviating ER stress.

### 3.7 CCDC85A promotes resistance of cancer cells to cisplatin

**Figure 7:**
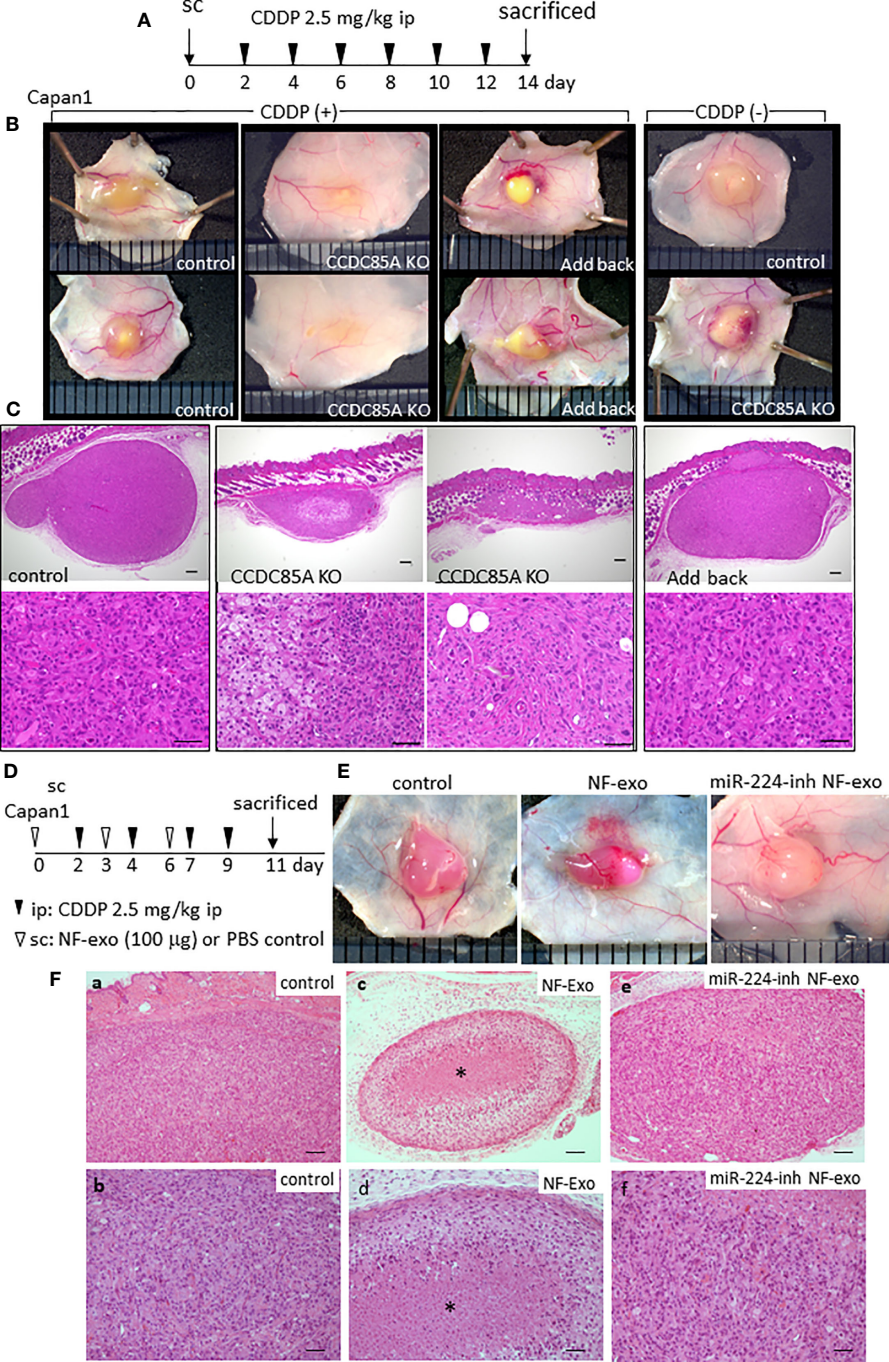
CCDC85A promotes the resistance of tumors against cisplatin treatment. **(A)** Capan1 control, CCDC85A^KO^, or addback cells were injected subcutaneously into 6-week-old nude mice and treated by CDDP as described in Materials and Methods, and sacrificed at day 14. **(B)** Representative appearance of the tumors. Tumors in mice without CDDP treatment were shown at the right. **(C)** Tumors were excised, fixed, and the maximum cut surface was subjected to H&E staining. Representative images are shown. Bar; 200 μm (upper panels), 50 μm (lower panels). Five mice were examined in each group. **(D)** Capan1 cells (1×10^6^) were subcutaneously injected with or without NF-exo (100 μg), and treated by CDDP as above. NF-exo was purchased as indicated by injecting into the tumor. **(E)** Representative images of tumors. miR-224-inh NF-exo: Exosomes were collected from NFs transfected with miR-224-3p inhibitor. **(F)** H&E staining of the maximum cut surface of each tumor, as described in **(C)** Bar; 100 μm (upper panels), 50 μm (lower panels). Bottom panels (b, d, f) are enlarged images of a, c, e, respectively. Asterisk indicates the nest of dead cancer cells. Five mice were examined per group, and representative results are shown.

The effect of CCDC85A on tumor growth upon treatment with CDDP was evaluated by subcutaneous injection of Capan1 cells into nude mice (**Figure 7A**). In the absence of CDDP, the size of control and CCDC85A^KO^ Capan1 tumors was similar (**Figure 7B**, right panel). However, after repeated injection of CDDP, CCDC85A^KO^ tumors were smaller than control tumors; this phenomenon was prevented by addback of CCDC85A to KO cells (**Figure 7B**, **S8A, B**). Histologically, CDDP-treated CCDC85A^KO^ cells appeared to be apoptotic, accompanied frequently by fibrosis and accumulation of macrophages; this was rarely observed in control Capan1 tumors (**Figure 7C**, **S8C**). Expression of CCDC85A in these tumors was confirmed by immunohistochemistry (**Figure S8D**). These observations suggest that CDDP is more cytotoxic to CCDC85A^KO^ cancer cells.

Similar results were obtained when using U87MG cells. CDDP-mediated reductions in tumor size were more evident in CCDC85A-miR U87MG cells (**Figure S9A, B**). Next, we examined the effects of exosomes containing miR-224-3p on tumor viability in the presence of CDDP. NF-derived exosomes (NF-exo), which contain miR-224-3p (**Figure S4D**), impaired resistance of cultured Capan1 cells to ER stress induced by TG, Tun, or CDDP treatment, which was rescued by pretreatment of NFs with a miR-224-3p inhibitor (**Figure S9C**). Capan1 cells were injected subcutaneously into mice with or without NF-exo, and NF-exo were injected into the tumor periodically (**Figure 7D**). Cancer cell death upon CDDP treatment was more evident in tumors exposed to NF-exo (**Figures 7E, Fa–d**); this was also rescued, at least partially, by pretreatment of NFs with an miR-224-3p inhibitor (**Figures 7E, Fe, f**).

Taken together, these results indicate that CCDC85A prevents apoptosis of cancer cells due to ER stress, thereby increasing resistance to CDDP.

## 4 Discussion

In this study, we focused on CCDC85A as a target of miR-224-3p. We show that CCDC85A is upregulated in tumors, and that this upregulation is modulated, at least in part, by miR-224-3p derived from exosomes secreted by fibroblasts. Therefore, expression of CCDC85A in cancer cells during the early stages of tumor progression may be suppressed by surrounding NF, or at the stage where cancer cells meet NF at the tumor periphery; during the advanced stages, this suppression is thought to be weaker and accompanied by CAF generation, as observed in **Figure 3A**. However, we cannot rule out at present the possibility that CCDC85A expression of cancer cells is not regulated exclusively by miRNA of fibroblasts, because in some tumor specimens, CCDC85A was stained in cancer cells but not in stromal fibroblasts.

Pancancer analysis of miR-224-3p and CCDC85A expression in the TCGA dataset using cBioportal revealed that deletion of miR-224-3p occurred in around 5.6% of cancers (**Figure S10A**). By contrast, high expression of CCDC85A mRNA was detected in around 6% of cancers, and higher incidence of CCDC85A expression was observed in some other cancers, e.g. thyroid cancer (**Figure S10B**). Future studies should try to evaluate both CCDC85A protein and miR-224-3p levels in cancer cells and stromal cells from various tumor specimens to generalize our conclusions, although an inverse relationship between CCDC85A protein and miR-224-3p was observed at least in some cancer cell lines (**Figure S10C**).

Although CCDC85A attenuates cell proliferation *in vitro* under standard conditions, it activates cell migration and increases resistance to ER stress, leading to resistance to cisplatin therapy. Regarding the mechanism underlying miR-224-3p-CCDC85A-mediated regulation of cell migration and invasion, we observed activation of Rac1/CDC42 by CCDC85A. Although the signaling pathway is not clear, CCDC85A promoted EMT (**Figure S3D**), which activates Rac1/CDC42 in various cancer cells (42, 43). Because CCDC85A upregulated TGFβ in cancer cells under the hypoxic conditions (**Figure S3F**), it may promote EMT and activate Rac1/CDC42. In addition, although the proliferative capacity of CCDC85A KO cells was higher than that of control cells under standard culture conditions, the tumor size in untreated mice was almost the same. This may also be dependent on differences in adaptation to hypoxic conditions *in vivo*.

Because retaining eIF2〈 phosphorylation is considered to exert a cytoprotective effect via prolonged arrest of global translation (44), it may be the reason for CCDC85A-mediated resistance to ER stress. In addition, expression of ATF4 (a downstream molecule of the PERK-eIF2〈 axis) is augmented by CCDC85A. ATF4 protects cells against ER stress by inducing autophagy; however, it also induces expression of proapoptotic CHOP when ER stress is unmitigated (45, 46). Although the mechanism that fine-tunes cell survival and apoptosis via the PERK pathway is not well understood, our results from the PERK inhibitor experiments indicate that CCDC85A-mediated activation of PERK-eIF2〈 signaling is cytoprotective against ER stress. In addition to the functions in ER, GRP78 and GRP94 migrate to the cell surface, where they act as neoantigens (41, 45, 46). Although we did not observe clear accumulation of CCDC85A on the cell surface, the role of CCDC85A outside the ER warrants further examination.

Our results suggest that tumors showing high expression of CCDC85A (e.g., gastric and pancreatic cancer) may be refractory to ER stress; therefore, treatments such as CDDP, which induce ER stress, are less effective than they are against CCDC85A-negative tumors. Future studies should examine the correlation between CCDC85A expression and drug resistance in matched-pair samples from patients receiving CDDP chemotherapy. Although the binding site is not fully determined at present, a peptide that blocks the CCDC85A-GRP78 or GRP94 interaction would prevent CCDC85A-mediated resistance to chemotherapy. To the best of our knowledge, this is the first study to examine the function of CCDC85A in tumors, and to identify miR-224 and CCDC85A as promising therapeutic targets.

## Data availability statement

The datasets GSE236957 for this study can be found in the Gene Expression Omnibus (GEO) in National Center for Biotechnology Information (NCBI) (https://www.ncbi.nlm.nih.gov/geo/).

## Ethics statement

The studies involving human participants were reviewed and approved by Akita University Ethics Committee (approval number 1662, 2190 Akita, Japan). The patients/participants provided their written informed consent to participate in this study. The animal study was reviewed and approved by Committee for Ethics of Animal Experimentation (approval number a-1-3175, Akita, Japan).

## Author contributions

MT was responsible for the design of the project, acquisition and analyzing data, manuscript review and editing, and project administration. ST was responsible for performing experiments, collecting, and analyzing data, and writing the original manuscript. KT, GI, SK, and MU were responsible for performing experiments, collecting, and analyzing data. KY and MY were responsible for generating cell lines. AG and KI were responsible for analyzing data and manuscript review. All authors contributed to the article and approved the submitted version.

## Supplementary material

Supplementary Figure 1 Representative microRNAs differentially regulated in NF-37 and CAF37. **(A)** Expression of αSMA and E-cadherin was evaluated in CAF and NF by Western blot. **(B)** Left: Partial enlargement of the heat map shown in Figure 1A. Right: MicroRNA was purified from NF-50 and CAF-50 cells, and subjected to miRNA-sequencing analysis as described in Materials and methods. Heat map showing the most statistically significant miRNAs upregulated in NF-50. **(C)** Representative up-regulated or downregulated microRNAs in NF-37 cells compared to CAF-37 cells (more than 4.0-fold) by miRNA microarray analysis were shown. **(D)** RT-PCR of miRNA precursors was performed on CAFs and NFs isolated from three patients. Amplified products were analyzed by electrophoresis on polyacrylamide gels. Intensities of the bands were quantified, and expression of each miRNA precursor was normalized by the loading control, and expressed as the relative ratio to CAF. **(E, F)** The miR-224-3p targets were selected by picking up the upregulated genes in CAF-37 relative to NF-37 among the candidate genes predicted by TargetScan software **(E)** or miRmap software **(F)**. The top 11 genes are shown.

Supplementary Figure 2 Example of quantitation of cell invasion areas. **(A)** Quantitation of the cell invasion area using Figure 1E (CAF-37) and Figure 2H (TIG-1-20). The areas of invading cells in the gel (shown in black) were quantified by ImageJ software, as described in Materials and Methods. Bar, 500 μm. **(B)** Validations of miR-224-3p in TIG-1-20 cells by qRT-PCR using TaqMan microRNA assays. Right: Expression of miR-224-3p was compared in the parent or CCDC85A overexpressed TIG-1-20 cells. **(C)** TIG-1-20 fibroblasts overexpressing CCDC85A (two clones; OE #1 and OE #2, shown in **Figure 2F**), or control cells, were cultured under standard conditions. Photographs were taken 5 days after the cells reached confluence. Representative images are shown. No apparent transforming foci were observed. Bar, 100 μm. **(D)** Example of quantitation of the CCDC85A positive area using **Figure 3A** (a). Split color images of the immunohistochemical analysis were prepared by ImageJ software, and quantified the area as described in A.

Supplementary Figure 3 CCDC85A promotes EMT in hypoxic condition. **(A)** (a, b) Human gastric cancer specimens (two cases) were immunostained with antibodies specific for CCDC85A (red), cytokeratin 19 (CK19, a cancer cell marker, green), αSMA (a marker of CAF, white), and DAPI, as described in the Supplementary materials and methods. (c, d) In case #2, merged images of CCDC85A and CK19 (c) or αSMA (converted to green, d) were prepared using ZEN software. The CCDC85A-expressing area in cancer cells (c) or CAFs (d) was evaluated by measuring the colocalized yellow area using ImageJ software; the results are expressed as a percentage (%) of the colocalized area relative to the total area of CK19 (cancer cells) or αSMA (CAFs). Bar, 50 μm. **(B)** Control and CCDC85AKO Capan1 cells were subjected to flow cytometry analysis to investigate cell cycle status as described in materials and methods. **(C)** Transwell invasion assay. Cells were seeded onto a Matrigel-coated membrane and harvested after 16 h. Cells present on the bottom surface of the membrane were counted. The results are representative of three independent experiments, each performed in duplicate. **P*< 0.01. (D–F) Capan1 parent or CCDC85A^KO^ cells were cultured under hypoxic conditions (1.0% O2) for 36 h. (D) Representative images of each cell are shown. Bar, 50 μm. **(E, F)** Protein lysates were prepared for western blot analysis of EMT-related molecules **(E)** and TGFβ **(F)**. In F, cleaved mature TGFβ was detected after a longer exposure (arrowhead).

Supplementary Figure 4 Transfer of miR-224-3p via exosomes regulates CCDC85A mediated cell migration. **(A)** Western blot of CCDC85A in U87MG cells stably expressing CCDC85A miRNAs (two independent miRNAs as described in Materials and Methods) or the control miRNA. **(B)** U87MG cells stably expressing control miRNA or CCDC85A miRNAs were subjected for 3D gel invasion assay as described in the legend of Fig 1E. Superior views of the gels were shown in the top panels. Pictures were taken 5 days after seeding the indicated cells. Bar, 500 μm. **(C)** In vitro proliferation of U87MG cells expressing CCDC85A miRs was evaluated as in the legend of Fig 2G. Data points indicate the average results from three dishes. ***P*<0.01. **(D)** Validations of miR-224-3p in exosomes derived from CAF-37 or NF-37 cells by qRT-PCR using TaqMan microRNA assays, as described in Materials and Methods. **(E, F)** Capan1 cells were transfected with miR-224-3p inhibitor (+), or control inhibitor (-), and exosomes secreted from NF-37 or control PBS was added. **(E)** Expression of CCDC85A in Capan1 cells was examined by Western blot. **(F)** Transwell assay was performed, and cells that migrated to the bottom surface of the membrane were counted. Results represent three independent experiments. **P*<0.01. **(G)** Representative appearance of CCDC85A^KO^ Capan1 cells in **Figure 4E**, treated by TG with or without PERK inhibitor.

Supplementary Figure 5 CCDC85A alleviates ER-stress in U87MG cells. **(A)** Appearance of U87MG control (top panels) and CCDC85A miR cells (middle and bottom panels) exposed to tunicamycin (Tun) as indicated. (Right) Cells were fixed, and immunostained with anti-cleaved caspase3 (green) and phalloidin (red) as indicated above the panel. Merged images are shown. Bar, 40 μm. **(B, C)** Thapsigargin (TG) or tunicamycin (Tun) **(B)**, or cisplatin (CDDP) **(C)**, were added to the medium (containing 10% FBS) of U87MG control and CCDC85A miR cells, and incubated for 30 h. Protein lysates were prepared and subjected for Western blot analysis with indicated antibodies.

Supplementary Figure 6 MiR-224-3p aggravates ER-stress induced apoptosis in CAFs. **(A)** CAF50 cells (control or miRIDIAN miR-224-3p transfected) were treated by TG, Tun or CDDP, or left untreated for 30h. Protein lysates were prepared and subjected for Western blot analysis with indicated antibodies. **(B)** Capan1 cells and U87MG cells (control, CCDC85A^KO^ or CCDC85A-miR cells) were treated with TG or left untreated, and then subjected to immunoblotting to examine expression of the indicated inflammation-related molecules.

Supplementary Figure 7 CCDC85A inhibits association of GRP94 with PERK. **(A, B)** U87MG cells were cultured with or without thapsigargin for 15h. Cell lysates were immunoprecipitated by anti-GRP78 antibody **(A)**, anti-GRP94 antibody **(B)** or control mouse IgG, and coprecipitated CCDC85A was detected by anti-CCDC85A antibody (indicated by asterisk). **(C)** COS1 cells were transfected with plasmids encoding the indicated genes with HA- or Halo-tag at C-terminus. Cell lysates were subjected for immunoprecipitation (IP) of PERK, and co-precipitated CCDC85A was detected. The antibodies used for the detection of each gene product are described in brackets. Expression of transfected genes in cell lysates (input) are shown at bottom. **(D)** COS1 cells were transiently transfected with PERK, GRP94 together with increasing amount of CCDC85A (L, H). PERK was immunoprecipitated by Halo magnet beads as in Fig 6I, and co-precipitated GRP94 was detected by anti-HA antibody. **(E)** U87MG control or CCDC85A miR cells were subjected for immunoprecipitation using anti-GRP94 antibody or the control normal rabbit IgG. Co-precipitated PERK was detected by Western blot.

Supplementary Figure 8 Depletion of CCDC85A from Capan1 cells promotes cisplatin-induced tumor shrinkage. **(A)** The tumor size of Capan1control, CCDC85AKO and CCDC85A addback cells in CDDP treated mice were evaluated by quantifying the tumor area of the maximum cut surface in H&E stained specimens. Results were expressed as the relative ratio to the tumor of control cells. Five mice were examined in each group. **P*< 0.01. **(B)** Line plots depicting changes in tumor size (diameter; mm) in each group shown in **Figures 7A–C**. Cont: control, KO: CCDC85A^KO^, Add: CCDC85A addback. **(C)** The same specimens in **Figure 7-C** were immunostained with anti-αSMA (green, fibroblasts), antiF4/80 (red, macrophages), and anti-cleaved caspase3 (white) as indicated at the bottom. Bar, 100 μm. **(D)** Expression of CCDC85A in the tumor sections shown in **Figures 7A–C** was confirmed by immunohistochemistry. Bar, 50 μm.

Supplementary Figure 9 Decrease of CCDC85A expression in U87MG cells promotes tumor reduction by cisplatin. **(A, B)** U87MG control and CCDC85A miR cells were injected subcutaneously into 6-week-old nude mice and treated by CDDP as indicated, and sacrificed at day 14. **(A)** Representative appearance of the tumors are shown. CDDP (-): Tumors in mice without CDDP treatment. Tumor area was quantified in the paraffin sections of the maximum cut surface as above and expressed as the relative ratio to the tumor of control cells. Five mice were examined in each group. **P*< 0.01. **(B)** Line plots showing changes in tumor size (diameter; mm) in each group. Cont: control U87MG cells, miR: CCDC85A miR cells. **(C)** Capan1 cells (1 × 10^6^) were treated (or not) for 2 days with NF-exo or exosomes collected from NFs transfected with an miR-224-3p inhibitor (miR-224-inh NF-exo) (20 μg each) and then incubated for 26 h with TG, Tun, or CDDP, as indicated above the lanes. Cell lysates were prepared for western blot analysis to examine apoptosis and expression of CCDC85A.

Supplementary Figure 10 Analysis of expression of CCDC85A and miR-224-3p in cancers from databases. **(A, B)** Data for miR-224-3p and CCDC85A were obtained from The Cancer Genome Atlas (TCGA) database and analyzed using cBioportal. **(A)** Pancancer analysis of the frequency of mutations, and amplifications and deletions, in miR-224-3p. **(B)** Percentage of CCDC85A mRNA “ high” patients from pancancer analysis. **(C)** Expression of CCDC85A and miR-224-3p by various cancer cell lines. Expression of CCDC85A was examined by Western blot analysis (upper panels), and miR-224-3p was validated by qRT-PCR using TaqMan microRNA assays (bottom panels).
