# Supplementary material for: CCDC85A is regulated by miR-224-3p and augments cancer cell resistance to endoplasmic reticulum stress
Source: Front Oncol. 2023 Jul 18;13:1196546. doi: 10.3389/fonc.2023.1196546 (PMC10391547; doi:10.3389/fonc.2023.1196546)
Supplement: Supplementary file 1 [file DataSheet_1.pdf]

Supplementary materials and methods

Plasmids, antibodies, and reagents

The cDNA constructs encoding human GRP78, GRP94, PERK and ATF6 were amplified by RT-PCR from the mRNA of 37CAF (CCDC85A) or 293T, and sequenced. Plasmid DNA encoding human ccdc85a (KIAA1912, corresponding to variant 2, 553 amino acids) was purchased from Kazusa Genome Technologies (Chiba, Japan). Deletion mutant forms of PERK (PERK-N; aa 1-535, PERK-C; aa 536-1116) were generated by PCR-based techniques. For expression in mammalian cells, cDNAs were tagged with either hemagglutinin (HA, 9 aa) or Halo (297 aa) at the C-terminus, and cloned into pCS2+ (Addgene, Watertown, MA, USA) or pFC14K (Promega, Madison, WI, USA), respectively.

Purchased antibodies were as follows: CCDC85A (Aviva System Biology, San Diego, CA, USA), HA (BabCO, Richmond CA, USA), Halo (Promega), Rac1 (BD Biosciences Franklin Lakes, NJ USA), CDC42 (Proteintech, Rosemont IL, USA), α-tubulin (Sigma Aldrich, St. Louis, MO, USA), p-PERK (Phospho-Thr 982, Signalway Antibody, Greenbelt, MD, USA), HIF1α (GeneTex, Irvine, CA, USA), E-cadherin (BD Bioscience, Chicago, IL, U.S.). Slug, Cleaved caspase-3, Phospho-eIF2α (Ser51) and eIF2α were from Cell Signaling (Danvers, MA, USA). Phospho-IRE1 (Ser724) was from CUSABIO (Houston, TX. U.S.). Antibodies against PERK, GRP78, IRE1α, DnaJC3 and ATF-4 were from Santa Cruz (Dallas, TX, USA). Rabbit IgG control polyclonal antibody was purchased from Proteintech (30000-0-AP). 1,1'-dioctadecyl-3,3,3',3'-tetramethyllindo-carbocyanine perchlorate (DiI) was purchased from Thermo Fisher Scientific (Waltham, MA USA). Thapsigargin, tunicamycin and cisplatin (CDDP) were purchased from FUJIFILM Wako pure chemical. Cell Proliferation ELISA, BrdU colorimetric detection kit was from Roche. miRIDIAN miR-224-3p (C-300734-05-0005), miR-224-3p-hairpin inhibitor (IH-301304-01-0002) and hairpin inhibitor negative control (IN-001005-01-05) were purchased from Dharmacon (Lafayette, CO, USA). PERK inhibitor I was purchased from Sigma Aldrich (GSK2606414). Matrigel was from BD Science.

Patient information of CAF/NF used in this study

The age (years), gender (M, F) and histological type of the gastric cancer patients corresponding to each CAF and NF cells are as follows.

CAF-37 / NF-37: 73 years-old, M, Diffuse-type (scirrhous)

CAF-50 / NF-50: 61 years-old, M, Intestinal-type (tubular)

CAF-58 / NF-58: 60 years-old, M, Diffuse-type (scirrhous)

Transfection and immunoprecipitation

Transfection of the plasmid DNA was performed by FuGene HD (Promega) for COS1 cells, and Lipofectamine 2000 (Invitrogen) for other cell lines, according to the manufacturer's instructions. Transfection of miRIDIAN miR-224-3p and miR-224-3p inhibitor was performed by RNAiMAX (Invitrogen). To immunoprecipitate the proteins, cell lysates were precleared with protein-G-agarose for 1 h, and 1 µg of antibody was incubated with 500 µg of cell lysate for 2 h at 4°C, and then precipitated with protein-G-agarose for 1 h at 4°C. Immunoprecipitates were extensively washed with PLC buffer, separated by SDS-PAGE, and immunoblotted. In some experiments, proteins tagged with HA or Halo (Promega) were expressed in COS1 cells, and the Halo-tagged protein was immunoprecipitated by Magne HaloTag Beads (Promega) and the co-precipitated HA-tagged protein was detected by anti-HA antibody. In some experiments, HRP-conjugated protein G was used as a secondary antibody to reduce signals from denatured IgG.

Rac1-GTP and CDC42-GTP pulldown assay

The activation of Rac1 or CDC42 was monitored by affinity precipitation of GTP-bound Rac1 or CDC42 with a GST-fusion of the p21-binding domain of PAK1 (GST-PBD) [1]. Briefly, cell lysates were prepared in lysis buffer [50mM HEPES (pH 7.5), 150mMNaCl, 10mM $MgCl_2$, 10% glycerol, 100mM NaF, 1mM Na_3VO_4 and 1% Triton X-100], and then incubated for 45 min at 4°C with gluthathione-Sepharose beads containing GST-PBD. Precipitates were washed four times in the same buffer, and the precipitated GTP-bound Rac1 or CDC42 was detected by immunoblotting.

3D gel invasion assay

The assay was performed as described previously [2]. Briefly, 200 µL of serum-free gel containing 2.25 mg/mL type-I collagen and 2.5 mg/mL Matrigel (BD Biosciences, Franklin Lakes, NJ, USA) was laid onto the upper chambers of Transwells in 24-well plates. Cells were labeled with DiI in accordance with the manufacturer's instructions. Fixed gels were observed under a confocal microscope (LSM780, Zeiss, Oberkochen, Germany). The area of invading cells was quantified using the ImageJ software (NIH) as demonstrated in Figure S2. The invasion index (I) was calculated as the area ratio of the objective cells to the control cells.

Transwell assay

Migration and invasion assays were performed using transwell chambers with a polycarbonate nucleopore membrane (8 μm pore size, BD Falcon). In invasion assay, the upper surface of transwell membrane was coated by matrigel (BD Bioscience) according to the manufactures' instructions. Test cells (2×10^4) in medium containing 0.2% FBS were seeded into the upper chambers of the transwells. The lower compartments were filled with the same medium supplemented with 10% FBS. Moved cells on the lower surface of the filter were fixed and stained with Giemsa's stain solution. The total number of migrating or invading cells was determined by counting cells in five microscopic fields per well, and expressed as the average number of cells per well. The assays were performed three times.

Immunoblotting

Cell lysates were prepared in PLC buffer [50mM HEPES (pH 7.5), 150mM NaCl, 1.5mM MgCl2, 1mM EGTA, 10% glycerol, 100mM NaF, 1mM Na3VO4, and 1% Triton X-100] containing protease inhibitors. Cell lysates were separated by SDS-PAGE, and immunoblotted. In some experiments, intensities were quantified using the ImageJ software.

5-Bromo-2'-Deoxyuridine (BrdU) incorporation

Cell proliferation was quantified by Cell proliferation ELISA kit, BrdU colorimetric (Roche, Basel. Switzerland) according to the manufacturer's instructions. In brief, cells were plated onto 96-well plates (1×10^4 cells/well) 48 hs before the addition of BrdU. Cells were reincubated for 6 hs and incorporated BrdU was detected with peroxidase-labeled anti-BrdU antibody and developed with tetramethyl-benzidine as a chromogenic substrate. The absorbance of the samples was measured at the wavelength of 450 nm using a microplate reader (Multiskan Fc, Thermo).

Immunohistochemical analysis

Paraffin blocks were sectioned and subjected to immunohistochemical staining using the Envision reagent (Dako, Santa Clara, CA, USA). Antigen retrieval was performed by placing sections in Target retrieval solution (Dako) and heating to 95°C in a water bath, according to the manufacturer's instructions. In co-immunostaining experiments, sections were sequentially stained with each antibody using an OpalTM four-color IHC Kit and fluorescently conjugated tyramide according to the manufacturer's instructions (PerkinElmer, Waltham, MA, U.S.). Horseradish peroxidase (HRP)-conjugated secondary antibody (GE Healthcare, Chicago, IL, USA) was added for 10 minutes, and

incubated with Opal kit working solution including the desired fluorophore. Tissues underwent the microwave treatment for removal of primary and secondary antibodies before another round of staining according to the Opal Multiplex IHC Assay Development Guide and Image Acquisition Information (Akoya Biosciences, Tokyo, Japan). Stained sections were observed under a confocal microscope (LSM780, Zeiss), and analyzed by Zen software (Zeiss).

1 Otsuki Y, Tanaka M, Yoshii S, Kawazoe N, Nakaya K, Sugimura H. Tumor metastasis suppressor nm23H1 regulates Rac1 GTPase by interaction with Tiam1. *Proc Natl Acad Sci U S A* 2001; 98: 4385-4390.

2 Satoyoshi R, Kuriyama S, Aiba N, Yashiro M, Tanaka M. Asporin activates coordinated invasion of scirrhous gastric cancer and cancer-associated fibroblasts. *Oncogene* 2015; 34: 650-660.
