# Supplementary material for: CCDC85A is regulated by miR-224-3p and augments cancer cell resistance to endoplasmic reticulum stress
Source: Front Oncol. 2023 Jul 18;13:1196546. doi: 10.3389/fonc.2023.1196546 (PMC10391547; doi:10.3389/fonc.2023.1196546)
Supplement: Supplementary Figure 1 — Representative microRNAs differentially regulated in NF-37 and CAF37. (A) Expression of αSMA and E-cadherin was evaluated in CAF and NF by Western blot. (B) Left: Partial enlargement of the heat map shown in Figure 1A. Right: MicroRNA was purified from NF-50 and CAF-50 cells, and subjected to miRNA-sequencing analysis as described in Materials and methods. Heat map showing the most statistically significant miRNAs upregulated in NF-50. (C) Representative up-regulated or downregulated microRNAs in NF-37 cells compared to CAF-37 cells (more than 4.0-fold) by miRNA microarray analysis were shown. (D) RT-PCR of miRNA precursors was performed on CAFs and NFs isolated from three patients. Amplified products were analyzed by electrophoresis on polyacrylamide gels. Intensities of the bands were quantified, and expression of each miRNA precursor was normalized by the loading control, and expressed as the relative ratio to CAF. (E, F) The miR-224-3p targets were selected by picking up the upregulated genes in CAF-37 relative to NF-37 among the candidate genes predicted by TargetScan software (E) or miRmap software (F). The top 11 genes are shown. [file DataSheet_2.pdf]

Fig S1

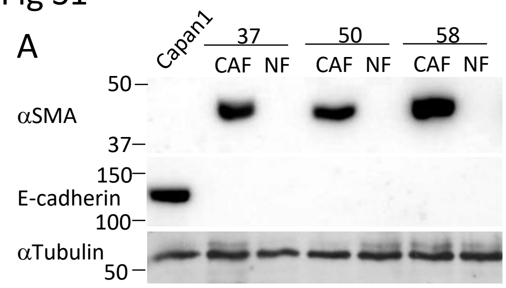

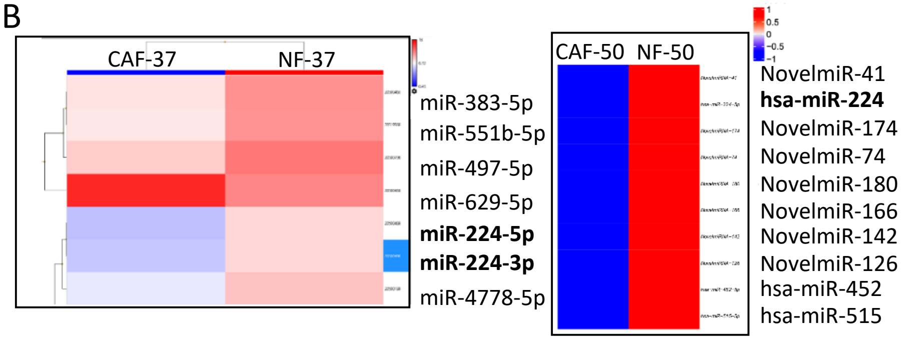

C

List of miRNA up or downregulated in NF

NF/CAF >4.0

fold	miRNA
18.74449	hsa-miR-383-5p
9.3712516	hsa-miR-551b-5p
7.1178752	hsa-miR-497-5p
6.2612939	hsa-miR-629-5p
5.872683	hsa-miR-224-5p
5.0962708	hsa-miR-224-3p
4.444283	hsa-miR-4778-5p
4.2618393	hsa-miR-4726-5p
4.1643266	hsa-miR-195-5p
4.1577288	hsa-miR-29b-2-5p
4.1018437	hsa-miR-4448

NF/CAF <0.25

fold	miRNA
0.0488314	hsa-miR-708-5p
0.0541047	hsa-miR-137
0.0951095	hsa-miR-6864-5p
0.1569637	hsa-miR-218-5p
0.1844549	hsa-miR-10a-3p
0.196761	hsa-miR-10a-5p
0.2088752	hsa-miR-34c-5p
0.2353272	hsa-miR-145-3p

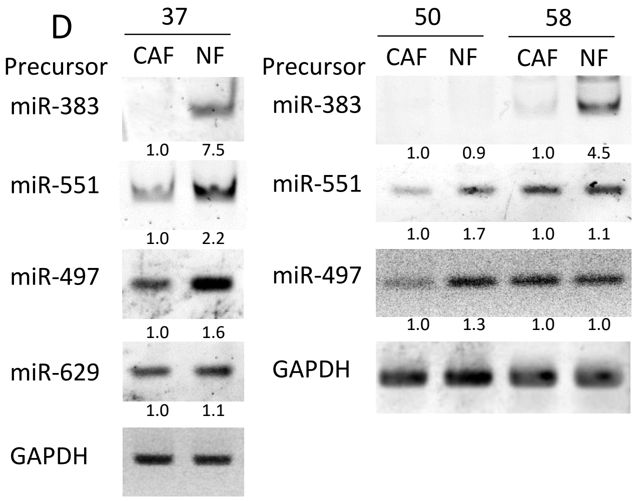

E Candidate target genes of miR-224-3p (TargetScan)

GeneSymbol	GeneName	Fold change (37T)vs.(37N)
CCDC85A	coiled-coil domain containing 85A	93.974
PKP2	plakophilin 2	92.858
HHIP	hedgehog interacting protein	92.436
CADM1	cell adhesion molecule 1	86.692
NCAM1	neural cell adhesion molecule 1	62.271
FRMD5	FERM domain containing 5	35.054
TYRP1	tyrosinase-related protein 1	32.893
HAPLN1	hyaluronan and proteoglycan link protein 1	31.600
ZMAT4	zinc finger, matrin-type 4	27.070
CLEC14A	C-type lectin domain family 14, member A	24.847
FRMD5	FERM domain containing 5	24.723
HTR2A	5-hydroxytryptamine (serotonin) receptor 2A, G protein-coupled	21.437

F Candidate target genes of miR-224-3p (miRmap)

GeneSymbol	GeneName	Fold change (37T)vs.(37N)
NRK	Nik related kinase	181.9731
CCDC85A	coiled-coil domain containing 85A	93.97387
PKP2	plakophilin 2	92.85847
HHIP	hedgehog interacting protein	92.43613
CADM1	cell adhesion molecule 1	86.69161
ASPN	asporin	75.70598
SDR42E1	short chain dehydrogenase/reductase family 42E, member 1	66.9553
NCAM1	neural cell adhesion molecule 1	62.27068
PLCXD3	phosphatidylinositol-specific phospholipase C, X domain containing 3	50.32256
ADRA2A	adrenergic, alpha-2A-, receptor	47.49184
FRMD5	FERM domain containing 5	35.05421

Fig S2

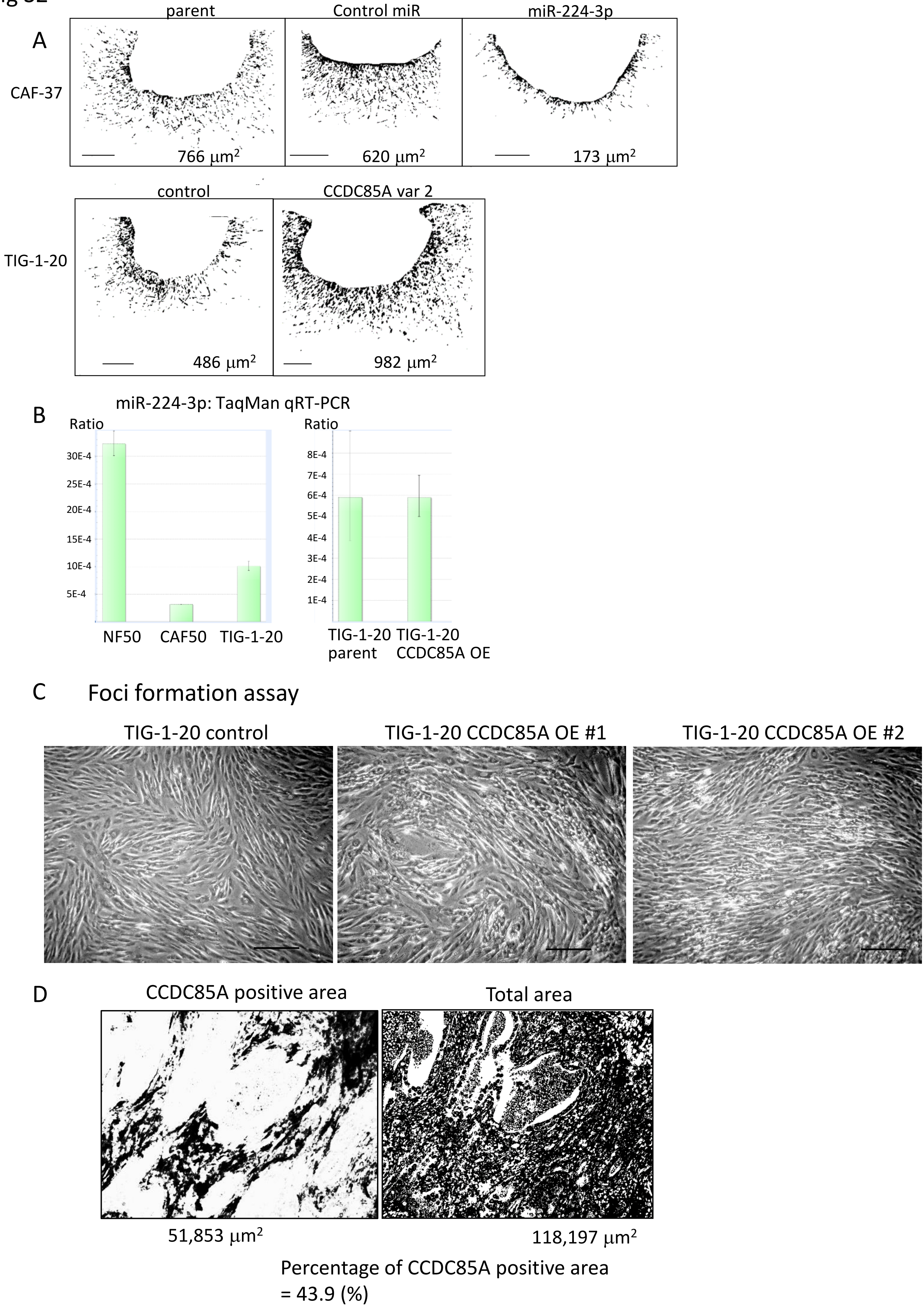
A
parent
Control miR
miR-224-3p
CAF-37
766 μm²
620 μm²
173 μm²
control
CCDC85A var 2
TIG-1-20
486 μm²
982 μm²
B
miR-224-3p: TaqMan qRT-PCR
Ratio
30E-4
25E-4
20E-4
15E-4
10E-4
5E-4
NF50
CAF50
TIG-1-20
Ratio
8E-4
7E-4
6E-4
5E-4
4E-4
3E-4
2E-4
1E-4
TIG-1-20 parent
TIG-1-20 CCDC85A OE
C
Foci formation assay
TIG-1-20 control
TIG-1-20 CCDC85A OE #1
TIG-1-20 CCDC85A OE #2
D
CCDC85A positive area
Total area
51,853 μm²
118,197 μm²
Percentage of CCDC85A positive area
= 43.9 (%)

Fig S3

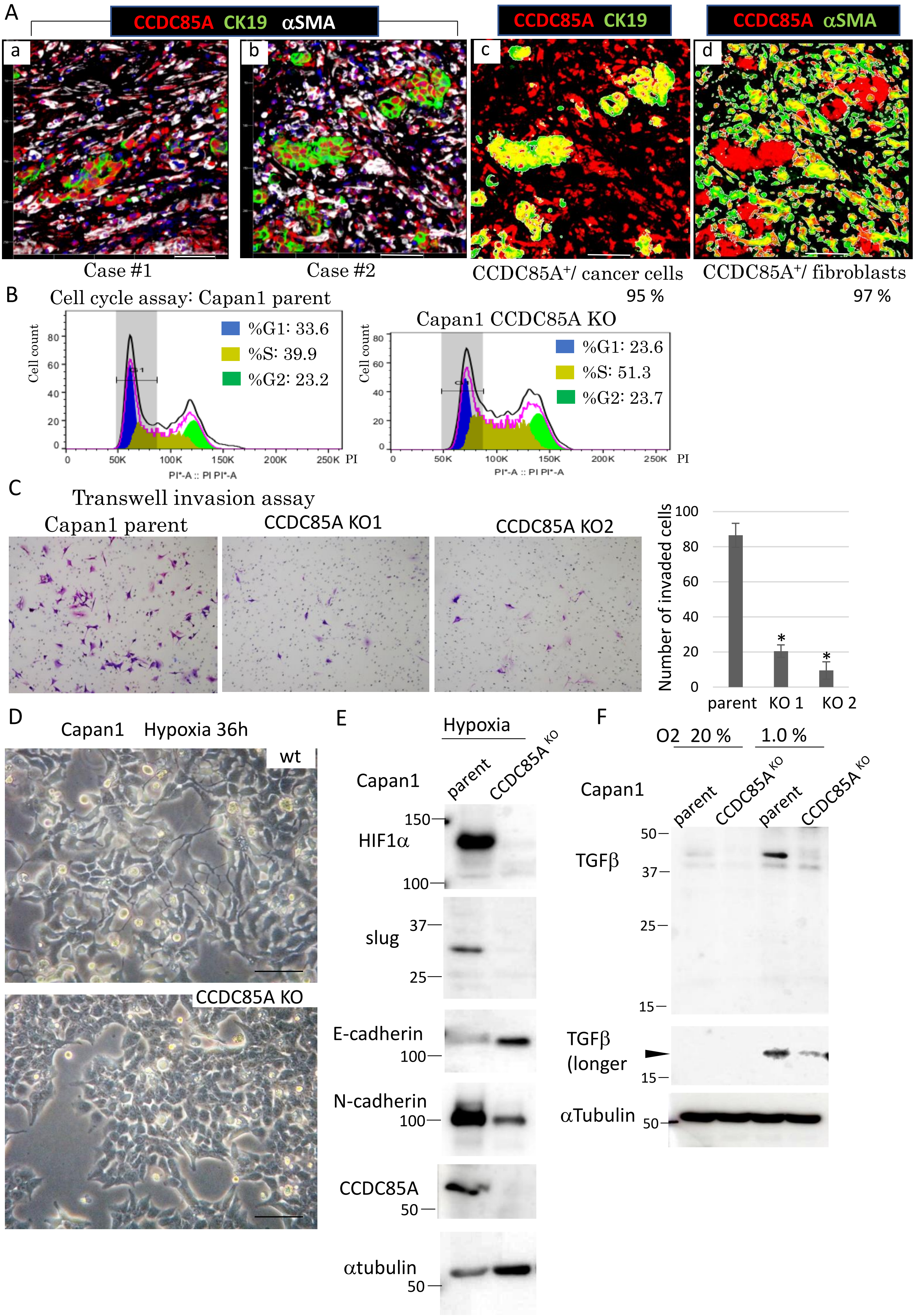

A
CCDC85A CK19 αSMA
CCDC85A CK19
CCDC85A αSMA
a
b
c
d
Case #1
Case #2
CCDC85A+/ cancer cells
95 %
CCDC85A+/ fibroblasts
97 %
B
Cell cycle assay: Capan1 parent
Cell count
%G1: 33.6
%S: 39.9
%G2: 23.2
PI*-A :: PI PI*-A
Capan1 CCDC85A KO
%G1: 23.6
%S: 51.3
%G2: 23.7
PI
C
Transwell invasion assay
Capan1 parent
CCDC85A KO1
CCDC85A KO2
Number of invaded cells
parent
KO 1
KO 2
D
Capan1 Hypoxia 36h
wt
CCDC85A KO
E
Hypoxia
Capan1
parent
CCDC85A KO
HIF1α
slug
E-cadherin
N-cadherin
CCDC85A
αtubulin
F
O2 20 %
1.0 %
Capan1
TGFβ
TGFβ (longer
αTubulin

Fig S4

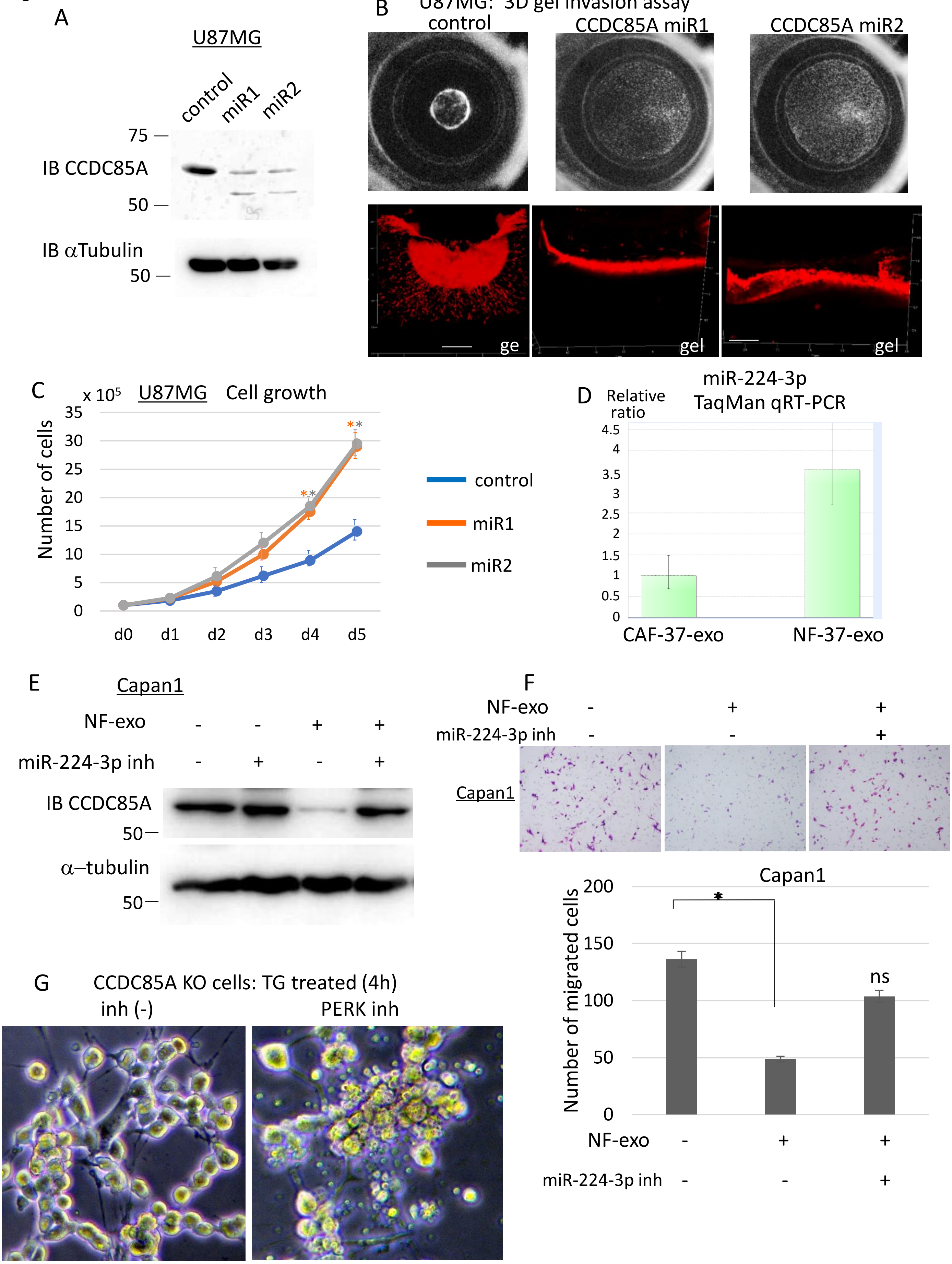
A
U87MG
control
miR1
miR2
75
IB CCDC85A
50
IB αTubulin
50
B
U87MG: 3D gel invasion assay
control
CCDC85A miR1
CCDC85A miR2
ge
gel
gel
C
x 10⁵
U87MG
Cell growth
Number of cells
35
30
25
20
15
10
5
0
d0
d1
d2
d3
d4
d5
control
miR1
miR2
D
Relative ratio
miR-224-3p
TaqMan qRT-PCR
4.5
4
3.5
3
2.5
2
1.5
1
0.5
0
CAF-37-exo
NF-37-exo
E
Capan1
NF-exo - - + +
miR-224-3p inh - + - +
IB CCDC85A
50
α–tubulin
50
F
NF-exo - + +
miR-224-3p inh - - +
Capan1
Capan1
Number of migrated cells
200
150
100
50
0
*
ns
NF-exo - + +
miR-224-3p inh - - +
G
CCDC85A KO cells: TG treated (4h)
inh (-)
PERK inh

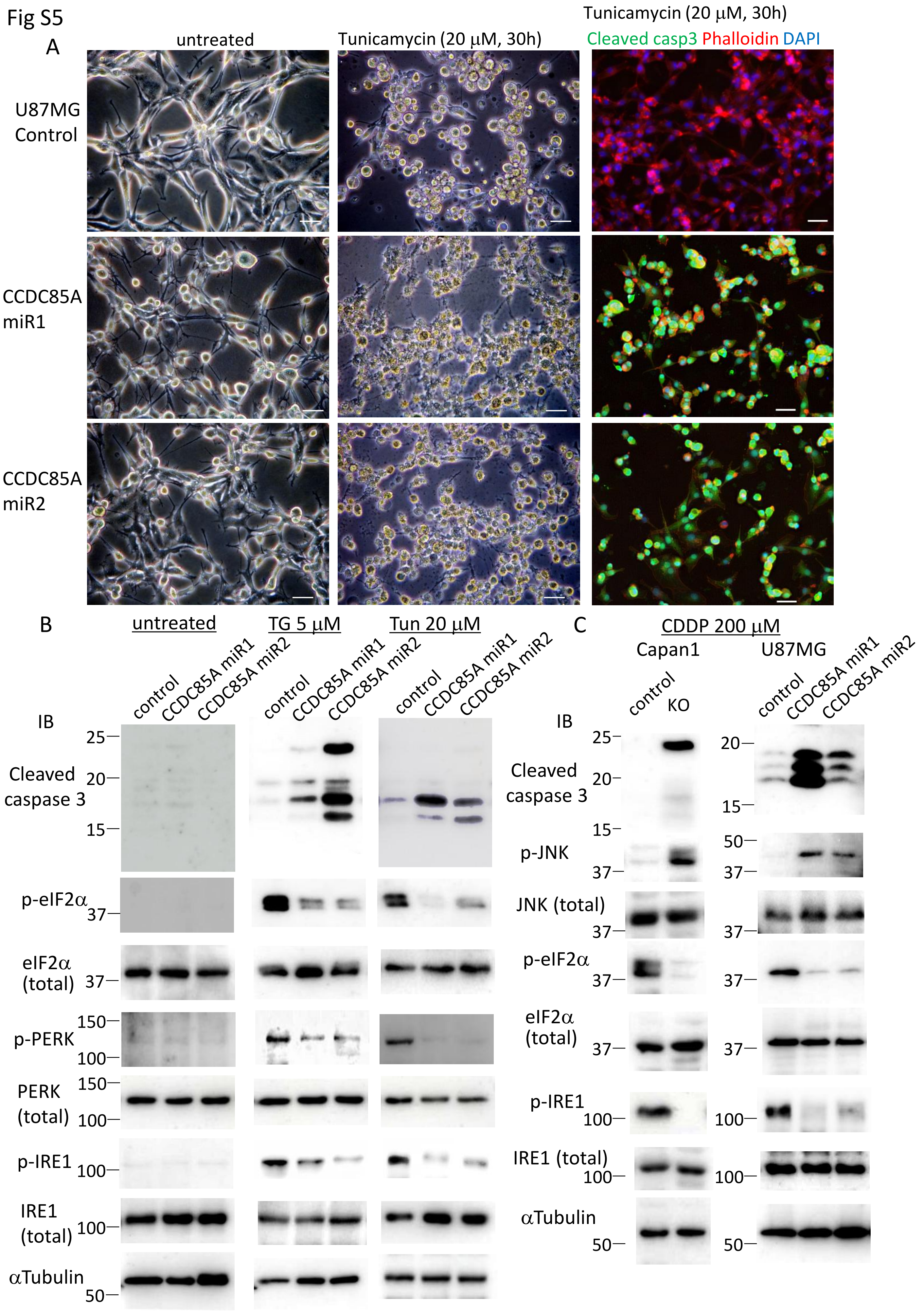
Fig S5
A
untreated
Tunicamycin (20 μM, 30h)
Tunicamycin (20 μM, 30h)
Cleaved casp3 Phalloidin DAPI
U87MG Control
CCDC85A miR1
CCDC85A miR2
B
untreated
TG 5 μM
Tun 20 μM
control
CCDC85A miR1
CCDC85A miR2
IB
Cleaved caspase 3
p-eIF2α
eIF2α (total)
p-PERK
PERK (total)
p-IRE1
IRE1 (total)
αTubulin
25
20
15
37
150
100
50
C
CDDP 200 μM
Capan1
U87MG
control
KO
p-JNK
JNK (total)

Fig S6

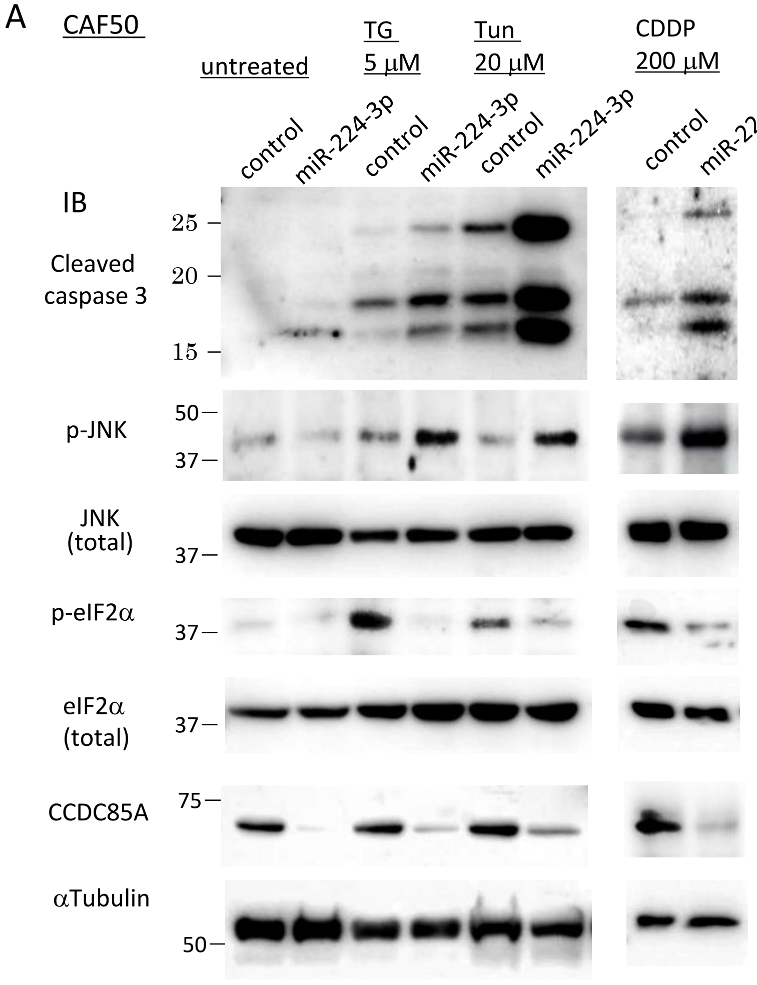
A
CAF50
untreated
TG 5 μM
Tun 20 μM
CDDP 200 μM
control
miR-224-3p
IB
Cleaved caspase 3
25
20
15
p-JNK
50
37
JNK (total)
p-eIF2α
eIF2α (total)
CCDC85A
75
αTubulin

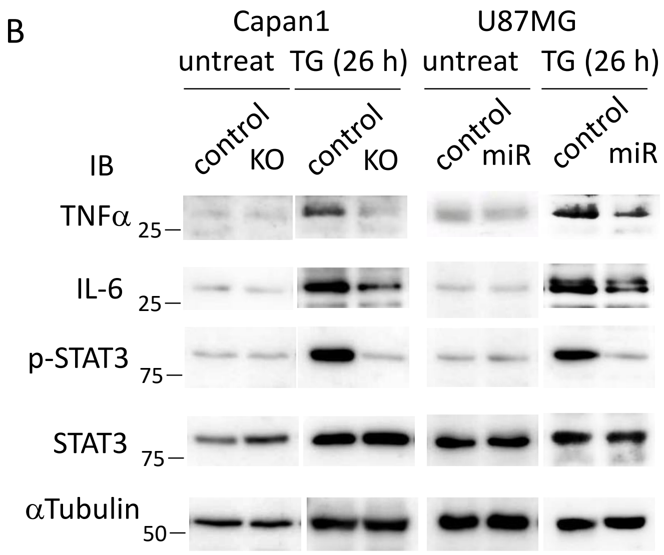
B
Capan1
U87MG
untreat
TG (26 h)
control
KO
miR
IB
TNFα
IL-6
p-STAT3
STAT3
αTubulin
25
75
50

Fig S7

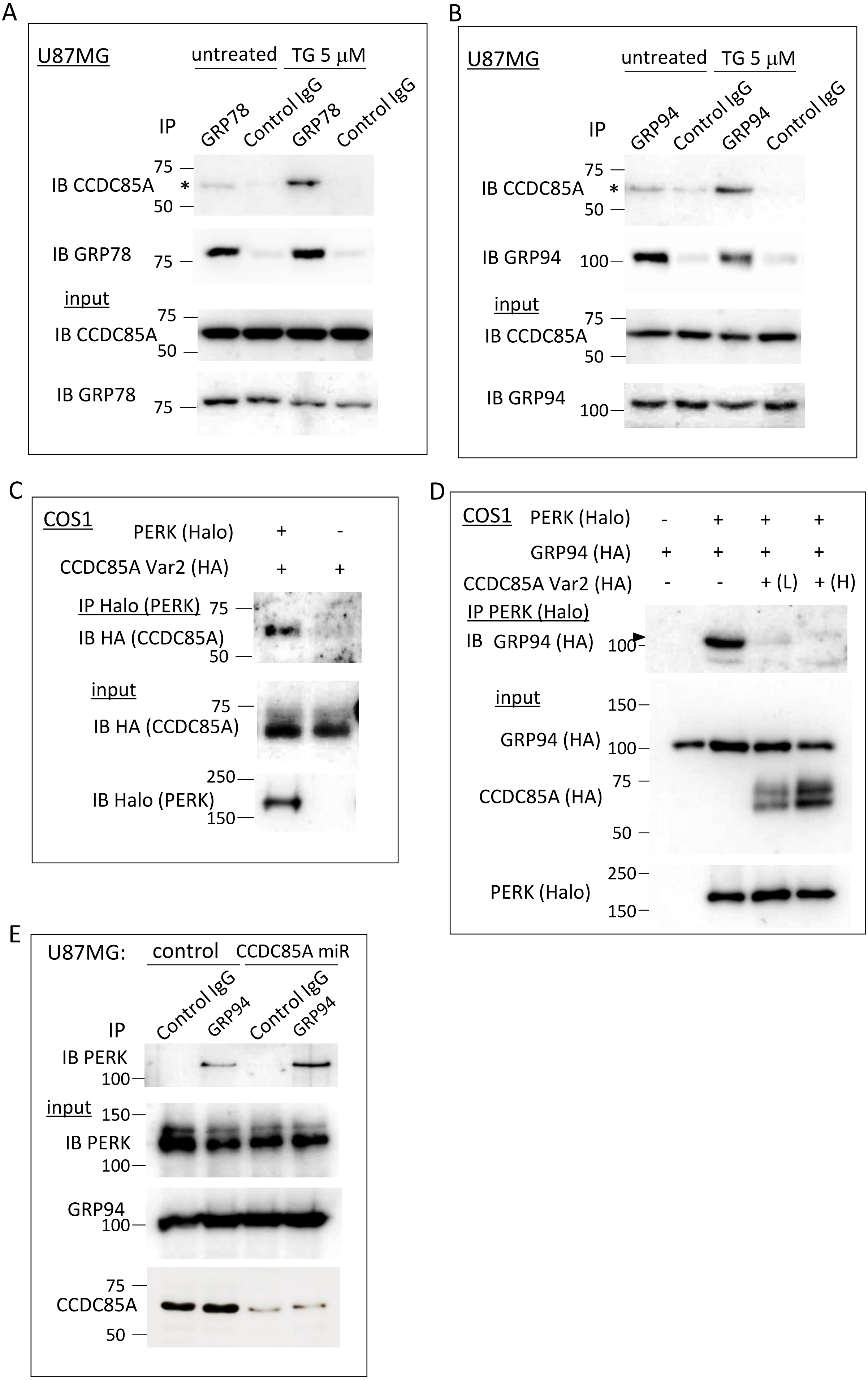

A
U87MG
untreated
TG 5 μM
IP
GRP78
Control IgG
GRP78
Control IgG
IB CCDC85A
*
75
50
IB GRP78
75
input
IB CCDC85A
75
50
IB GRP78
75
B
U87MG
untreated
TG 5 μM
IP
GRP94
Control IgG
GRP94
Control IgG
IB CCDC85A
*
75
50
IB GRP94
100
input
IB CCDC85A
75
50
IB GRP94
100
C
COS1
PERK (Halo) + -
CCDC85A Var2 (HA) + +
IP Halo (PERK)
IB HA (CCDC85A)
75
50
input
IB HA (CCDC85A)
75
IB Halo (PERK)
250
150
D
COS1
PERK (Halo) - + + +
GRP94 (HA) + + + +
CCDC85A Var2 (HA) - - + (L) + (H)
IP PERK (Halo)
IB GRP94 (HA)
100
input
150
GRP94 (HA)
100
75
CCDC85A (HA)
50
250
PERK (Halo)
150
E
U87MG:
control
CCDC85A miR
IP
Control IgG
GRP94
Control IgG
GRP94
IB PERK
100
input
150
IB PERK
100
GRP94
100
75
CCDC85A
50

Fig S8

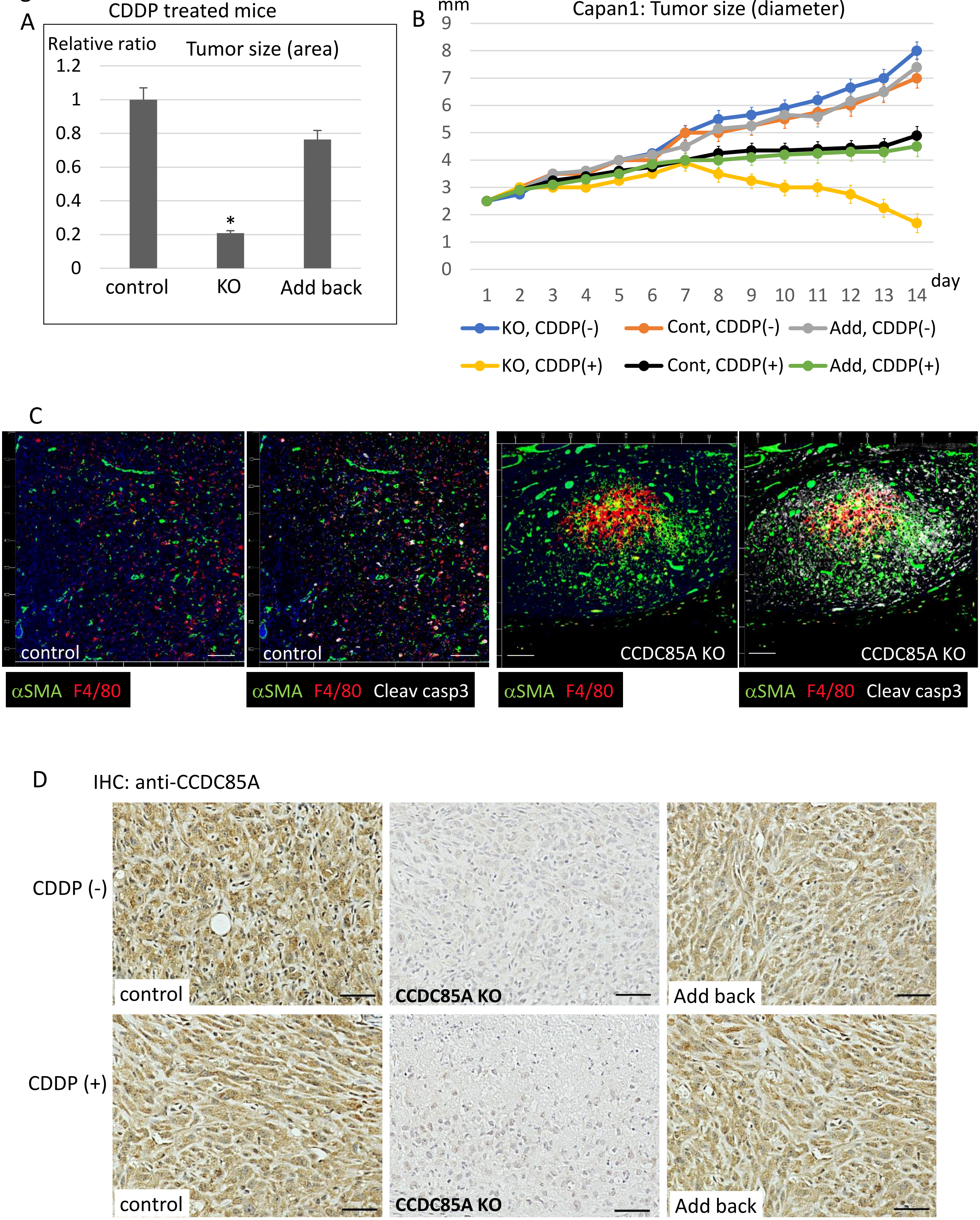

A
CDDP treated mice
Relative ratio
Tumor size (area)
1.2
1
0.8
0.6
0.4
0.2
0
*
control
KO
Add back
B
mm
Capan1: Tumor size (diameter)
9
8
7
6
5
4
3
2
1
0
1 2 3 4 5 6 7 8 9 10 11 12 13 14
day
KO, CDDP(-)
Cont, CDDP(-)
Add, CDDP(-)
KO, CDDP(+)
Cont, CDDP(+)
Add, CDDP(+)
C
control
control
CCDC85A KO
CCDC85A KO
αSMA F4/80
αSMA F4/80 Cleav casp3
αSMA F4/80
αSMA F4/80 Cleav casp3
D
IHC: anti-CCDC85A
CDDP (-)
CDDP (+)
control
CCDC85A KO
Add back
control
CCDC85A KO
Add back

Fig S9

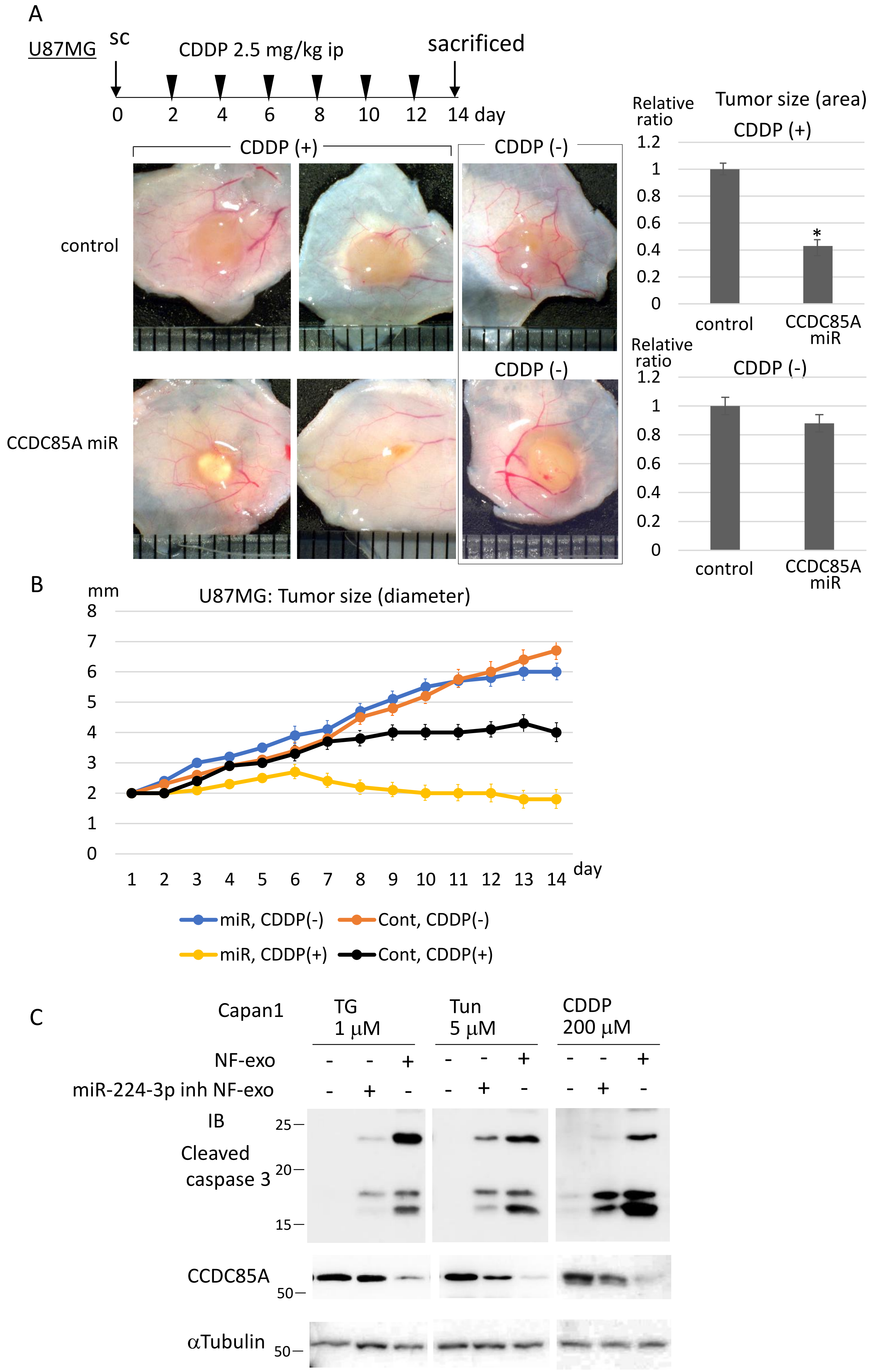
A
U87MG
sc
CDDP 2.5 mg/kg ip
sacrificed
0 2 4 6 8 10 12 14 day
CDDP (+)
CDDP (-)
control
CCDC85A miR
Relative ratio
Tumor size (area)
CDDP (+)
1.2 1 0.8 0.6 0.4 0.2 0
*
control
CCDC85A miR
Relative ratio
CDDP (-)
1.2 1 0.8 0.6 0.4 0.2 0
control
CCDC85A miR
B
mm
U87MG: Tumor size (diameter)
8 7 6 5 4 3 2 1 0
1 2 3 4 5 6 7 8 9 10 11 12 13 14
day
miR, CDDP(-)
Cont, CDDP(-)
miR, CDDP(+)
Cont, CDDP(+)
C
Capan1
TG 1 μM
Tun 5 μM
CDDP 200 μM
NF-exo - - + - - + - - +
miR-224-3p inh NF-exo - + - - + - - + -
IB
Cleaved caspase 3
25
20
15
CCDC85A
50
αTubulin
50

Fig S10

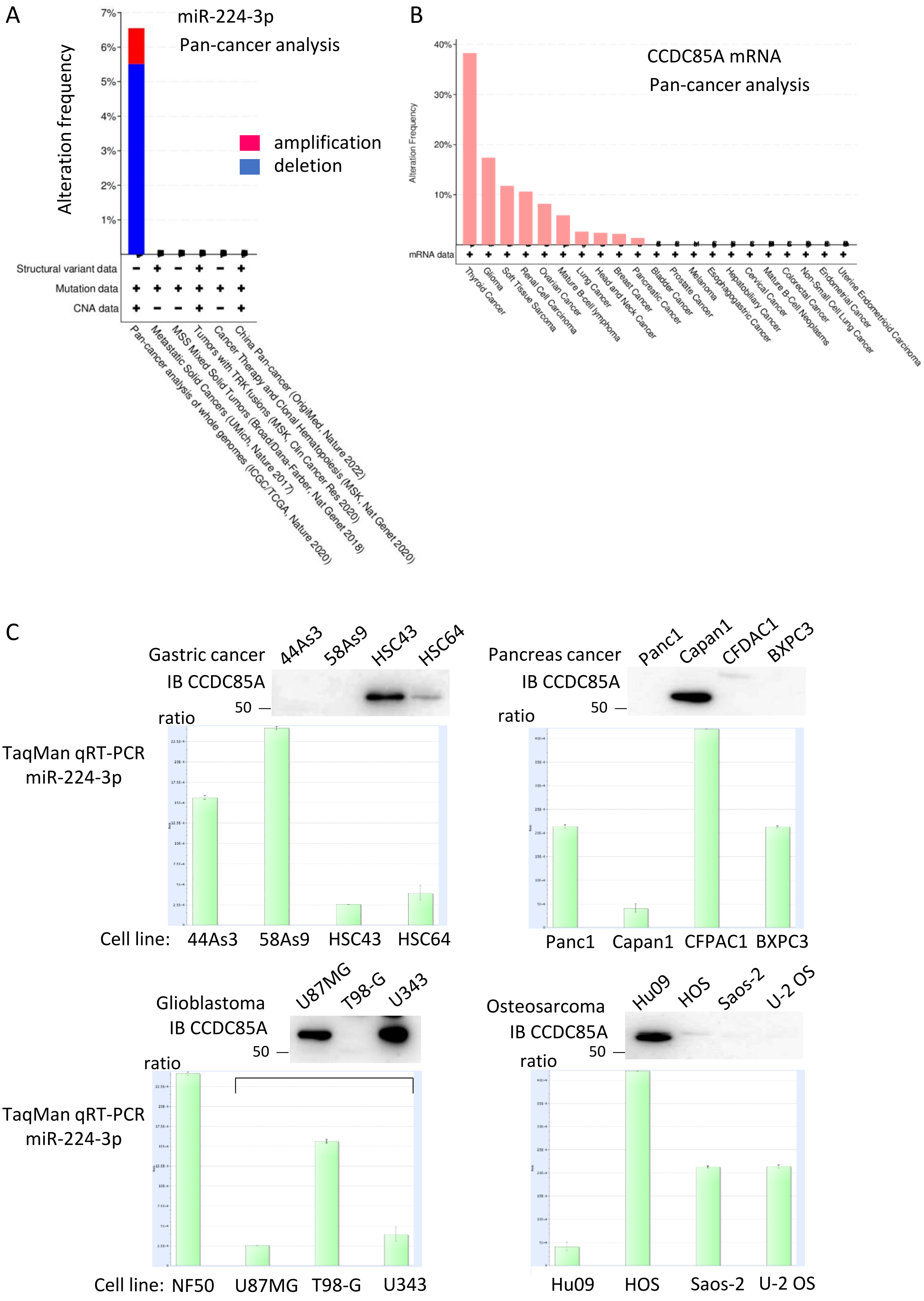

A
miR-224-3p
Pan-cancer analysis
Alteration frequency
7%
6%
5%
4%
3%
2%
1%
amplification
deletion
Structural variant data
Mutation data
CNA data
Pan-cancer analysis of whole genomes (ICGC/TCGA, Nature 2020)
Metastatic Solid Cancers (UMich, Nature 2017)
MSS Mixed Solid Tumors (Broad/Dana-Farber, Nat Genet 2018)
Tumors with TRK fusions (MSK, Clin Cancer Res 2020)
Cancer Therapy and Clonal Hematopoiesis (MSK, Nat Genet 2020)
China Pan-cancer (OrigiMed, Nature 2022)
B
CCDC85A mRNA
Pan-cancer analysis
Alteration Frequency
40%
30%
20%
10%
mRNA data
Thyroid Cancer
Glioma
Soft Tissue Sarcoma
Renal Cell Carcinoma
Ovarian Cancer
Mature B-cell lymphoma
Lung Cancer
Head and Neck Cancer
Breast Cancer
Pancreatic Cancer
Bladder Cancer
Prostate Cancer
Melanoma
Esophagogastric Cancer
Hepatobiliary Cancer
Cervical Cancer
Mature B-Cell Neoplasms
Colorectal Cancer
Non-Small Cell Lung Cancer
Endometrial Cancer
Uterine Endometrioid Carcinoma
C
Gastric cancer
44As3
58As9
HSC43
HSC64
IB CCDC85A
50
ratio
TaqMan qRT-PCR
miR-224-3p
Cell line: 44As3 58As9 HSC43 HSC64
Pancreas cancer
Panc1
Capan1
CFDAC1
BXPC3
IB CCDC85A
50
ratio
Panc1 Capan1 CFPAC1 BXPC3
Glioblastoma
U87MG
T98-G
U343
IB CCDC85A
50
ratio
TaqMan qRT-PCR
miR-224-3p
Cell line: NF50 U87MG T98-G U343
Osteosarcoma
Hu09
HOS
Saos-2
U-2 OS
IB CCDC85A
50
ratio
Hu09 HOS Saos-2 U-2 OS
